# Taxation of unprocessed sugar or sugar‐added foods for reducing their consumption and preventing obesity or other adverse health outcomes

**DOI:** 10.1002/14651858.CD012333.pub2

**Published:** 2020-04-09

**Authors:** Manuela Pfinder, Thomas L Heise, Michele Hilton Boon, Frank Pega, Candida Fenton, Ursula Griebler, Gerald Gartlehner, Isolde Sommer, Srinivasa Vittal Katikireddi, Stefan K Lhachimi

**Affiliations:** AOK Baden‐WürttembergDepartment of Health PromotionPresselstr. 19StuttgartBaden‐WürttembergGermany70191; University Hospital, University of HeidelbergDepartment of General Practice and Health Services ResearchVossstrasse 2HeidelbergBremenGermanyD‐69115; University of BremenInstitute for Public Health and Nursing Research, Health Sciences BremenBibliothekstr. 1BremenBremenGermany28359; Leibniz Institute for Prevention Research and EpidemiologyResearch Group for Evidence‐Based Public HealthAchterstr. 30BremenGermany28359; University of GlasgowMRC/CSO Social and Public Health Sciences UnitGlasgowUK; University of OtagoPublic Health23A Mein Street, NewtownWellingtonNew Zealand6242; University of EdinburghUsher Institute of Population Health Sciences and InformaticsMedical SchoolTeviot PlaceEdinburghUKEH8 9AG; Danube University KremsCochrane Austria, Department for Evidence‐based Medicine and EvaluationDr.‐Karl‐Dorrek Str. 30KremsAustria3500; University of BremenDepartment for Health Services Research, Institute for Public Health and Nursing Research, Health Sciences BremenBibliotheksstr. 1BremenGermany28359

## Abstract

**Background:**

Global prevalence of overweight and obesity are alarming. For tackling this public health problem, preventive public health and policy actions are urgently needed. Some countries implemented food taxes in the past and some were subsequently abolished. Some countries, such as Norway, Hungary, Denmark, Bermuda, Dominica, St. Vincent and the Grenadines, and the Navajo Nation (USA), specifically implemented taxes on unprocessed sugar and sugar‐added foods. These taxes on unprocessed sugar and sugar‐added foods are fiscal policy interventions, implemented to decrease their consumption and in turn reduce adverse health‐related, economic and social effects associated with these food products.

**Objectives:**

To assess the effects of taxation of unprocessed sugar or sugar‐added foods in the general population on the consumption of unprocessed sugar or sugar‐added foods, the prevalence and incidence of overweight and obesity, and the prevalence and incidence of other diet‐related health outcomes.

**Search methods:**

We searched CENTRAL, *Cochrane Database of Systematic Reviews*, MEDLINE, Embase and 15 other databases and trials registers on 12 September 2019. We handsearched the reference list of all records of included studies, searched websites of international organisations and institutions, and contacted review advisory group members to identify planned, ongoing or unpublished studies.

**Selection criteria:**

We included studies with the following populations: children (0 to 17 years) and adults (18 years or older) from any country and setting. Exclusion applied to studies with specific subgroups, such as people with any disease who were overweight or obese as a side‐effect of the disease. The review included studies with taxes on or artificial increases of selling prices for unprocessed sugar or food products that contain added sugar (e.g. sweets, ice cream, confectionery, and bakery products), or both, as intervention, regardless of the taxation level or price increase. In line with Cochrane Effective Practice and Organisation of Care (EPOC) criteria, we included randomised controlled trials (RCTs), cluster‐randomised controlled trials (cRCTs), non‐randomised controlled trials (nRCTs), controlled before‐after (CBA) studies, and interrupted time series (ITS) studies. We included controlled studies with more than one intervention or control site and ITS studies with a clearly defined intervention time and at least three data points before and three after the intervention. Our primary outcomes were consumption of unprocessed sugar or sugar‐added foods, energy intake, overweight, and obesity. Our secondary outcomes were substitution and diet, expenditure, demand, and other health outcomes.

**Data collection and analysis:**

Two review authors independently screened all eligible records for inclusion, assessed the risk of bias, and performed data extraction.Two review authors independently assessed the certainty of the evidence using the GRADE approach.

**Main results:**

We retrieved a total of 24,454 records. After deduplicating records, 18,767 records remained for title and abstract screening. Of 11 potentially relevant studies, we included one ITS study with 40,210 household‐level observations from the Hungarian Household Budget and Living Conditions Survey. The baseline ranged from January 2008 to August 2011, the intervention was implemented on September 2011, and follow‐up was until December 2012 (16 months). The intervention was a tax ‐ the so‐called 'Hungarian public health product tax' ‐ on sugar‐added foods, including selected foods exceeding a specific sugar threshold value. The intervention includes co‐interventions: the taxation of sugar‐sweetened beverages (SSBs) and of foods high in salt or caffeine.

The study provides evidence on the effect of taxing foods exceeding a specific sugar threshold value on the consumption of sugar‐added foods. After implementation of the Hungarian public health product tax, the mean consumption of taxed sugar‐added foods (measured in units of kg) decreased by 4.0% (standardised mean difference (SMD) −0.040, 95% confidence interval (CI) −0.07 to −0.01; very low‐certainty evidence).

The study was at low risk of bias in terms of performance bias, detection bias and reporting bias, with the shape of effect pre‐specified and the intervention unlikely to have any effect on data collection. The study was at unclear risk of attrition bias and at high risk in terms of other bias and the independence of the intervention. We rated the certainty of the evidence as very low for the primary and secondary outcomes.

The Hungarian public health product tax included a tax on sugar‐added foods but did not include a tax on unprocessed sugar. We did not find eligible studies reporting on the taxation of unprocessed sugar. No studies reported on the primary outcomes of consumption of unprocessed sugar, energy intake, overweight, and obesity. No studies reported on the secondary outcomes of substitution and diet, demand, and other health outcomes. No studies reported on differential effects across population subgroups.

We could not perform meta‐analyses or pool study results.

**Authors' conclusions:**

There was very limited evidence and the certainty of the evidence was very low. Despite the reported reduction in consumption of taxed sugar‐added foods, we are uncertain whether taxing unprocessed sugar or sugar‐added foods has an effect on reducing their consumption and preventing obesity or other adverse health outcomes. Further robustly conducted studies are required to draw concrete conclusions on the effectiveness of taxing unprocessed sugar or sugar‐added foods for reducing their consumption and preventing obesity or other adverse health outcomes.

## Plain language summary

**Does taxation of unprocessed sugar or foods with added sugar reduce their consumption and prevent obesity or other adverse health outcomes?**

**Why is this review important?**

As outlined by the World Health Organization, 'globesity' (the rise in overweight and obesity globally) is a major world challenge. A sugar‐rich diet, especially when combined with physical inactivity, may cause overweight and obesity, and other harmful health outcomes. There are direct costs to healthcare services of people being overweight or obese, such as preventing and treating health problems that this causes. There are also costs to society as a whole when people who are ill through being overweight or obese are unable to work.

**Who will be interested in this review?**

This review may be of interest to government public health agencies, policy decision‐makers, food retailers, and food industries. This review and subsequent updates of this review may change policy and affect a government's motivation to create a tax on unprocessed sugar and foods with added sugar. It also may motivate food industries to reformulate their products to contain lower levels of added sugar.

**What question does this review aim to answer?**

We wanted to know if taxation of unprocessed sugar and foods with added sugar (other than sugar‐sweetened beverages (SSBs)) reduced their consumption, changed people's energy intake, and reduced overweight and obesity. We also wanted to know if taxation changed people's diet and spending, and had an effect on other diet‐related health problems.

**Which studies were included in the review?**

We searched for ongoing or published studies up to October 2019. Of a total of 24,454 records retrieved, we identified one 'interrupted time series' (ITS) study meeting our eligibility criteria to assess the impact of a tax on sugar‐added foods (but not unprocessed sugar). The study used data from the Hungarian Household Budget and Living Conditions Survey, with observations from 40,210 households. Evidence from the study included a 'baseline' (the situation before taxation), ranging from January 2008 to August 2011. The Hungarian public health product tax was implemented on September 2011. The duration of the follow‐up period (measuring the effects of taxation) was 16 months. The study was funded by the Scottish Institute for Research in Economics (SIRE) Early Career Engagement Grant.

**What does the evidence from the review reveal?**

The included study provided very limited evidence that taxing foods with added sugar reduced their consumption by 4%. We are very uncertain about this evidence because the study did not use the strongest methods, looked at other kinds of taxation as well as taxing foods with added sugar, and may not have correctly classified food types. We are uncertain whether taxing foods with added sugar has an effect on reducing their consumption. The included study did not investigate the effects of taxing unprocessed sugar.

**What should happen next?**

Further research is needed to assess the effectiveness of taxing unprocessed sugar or foods with added sugar for reducing their consumption and preventing obesity or other adverse health outcomes. Studies should take place in countries that have implemented these taxes and should look at cost‐effectiveness as well as the health benefits of taxing unprocessed sugar or foods with added sugar as a public health policy for preventing overweight, obesity or other adverse health outcomes. Countries that have implemented these taxes are Bermuda, Dominica, Hungary, India, Norway, the Navajo Nation (USA), and St. Vincent and Grenadines.

## Summary of findings

**Summary of findings for the main comparison:** 'Summary of findings' table for primary outcomes: Taxation of sugar‐added foods compared to no taxation for reducing consumption of sugar‐added foods

**Taxation of sugar‐added foods compared to no taxation for reducing consumption of sugar‐added foods**				
**Population:** general population **Setting:** Hungary **Intervention:** taxation of sugar‐added foods **Comparison:** no taxation				
**Outcomes**	Anticipated absolute effects^*^ (95% CI)	**№ of participants (studies)**	**Certainty of the evidence (GRADE)**	**Comments**
**Mean consumption (purchased quantities) of taxed sugar‐added foods** Assessed with: Percentage change Follow‐up: 16 months	There was a decrease in the mean consumption of taxed sugar‐added foods by 4.0% (SMD −0.040, 95% CI −0.07 to −0.01) after implementation of the Hungarian public health product tax intervention. The effect is based on very low‐certainty evidence.	40,210 household‐level observations (1 observational study)	⊕⊝⊝⊝ **Very low**^a^	
**Consumption of unprocessed sugar**	See comment	0 (0)	See comment	Outcome not measured^b^
**Energy intake from unprocessed sugar or sugar‐added foods**	See comment	0 (0)	See comment	Outcome not measured^b^
**Total energy intake**	See comment	0 (0)	See comment	Outcome not measured^b^
**Overweight**	See comment	0 (0)	See comment	Outcome not measured^b^
**Obesity**	See comment	0 (0)	See comment	Outcome not measured^b^
**CI:** confidence interval; **SMD:** standardised mean difference				
**GRADE Working Group grades of evidence** **High certainty:** we are very confident that the true effect lies close to that of the estimate of the effect. **Moderate certainty:** we are moderately confident in the effect estimate: the true effect is likely to be close to the estimate of the effect, but there is a possibility that it is substantially different. **Low certainty:** our confidence in the effect estimate is limited: the true effect may be substantially different from the estimate of the effect. **Very low certainty:** we have very little confidence in the effect estimate: the true effect is likely to be substantially different from the estimate of effect.				

^a^Non‐randomised study (downgraded to low), downgraded one additional level (to very low) for risk of bias due to simultaneous intervention of other taxes and likely misclassification of food products as to whether taxed or untaxed. Certainty is also affected by indirectness because the Hungarian tax is related to specific sugar contents in the particular food categories that were taxed, and the study measured purchased quantities and not consumption. Thus, it is not a direct representation of the effect of a complete tax on sugar or sugar‐added foods. ^b^No study measured effects of taxing unprocessed sugar or sugar‐added foods on the consumption of unprocessed sugar, energy intake from unprocessed sugar or sugar‐added foods, total energy intake, overweight or obesity. *Effects are presented as SMDs as the number of distinct households and participants was not available to calculate valid MDs.

**Summary of findings 2:** 'Summary of findings' table for secondary outcomes: Taxation of sugar‐added foods compared to no taxation for reducing expenditure on and assessing substitution of sugar‐added foods

**Taxation of sugar‐added foods compared to no taxation for reducing expenditure on and assessing substitution of sugar‐added foods**				
**Population:** general population **Setting:** Hungary **Intervention:** taxation of sugar‐added foods **Comparison:** no taxation				
**Outcomes**	Anticipated absolute effects^*^ (95% CI)	**№ of participants (studies)**	**Certainty of the evidence (GRADE)**	**Comments**
**Substitution: mean consumption (purchased quantities) of untaxed sugar‐added foods** Assessed with percentage change Follow‐up: 16 months	There was no direct substitution effect. The mean consumption of untaxed sugar‐added foods even decreased after the implementation of the tax by 1.3% (SMD −0.013, 95% CI −0.05 to 0.02). The effect is based on very low‐certainty evidence.	40,210 household‐level observations (1 observational study)	⊕⊝⊝⊝ **Very low**^a^	
**Substitution: difference in mean consumption (purchased quantities) of untaxed sugar‐added foods compared with untaxed sugar‐added foods** Assessed with percentage change Follow‐up: 16 months	The mean consumption of taxed sugar‐added foods differed from the mean consumption of untaxed sugar‐added foods after the implementation of the intervention by 2.8% (SMD −0.028, 95% CI −0.07 to 0.02). The effect is based on very low‐certainty evidence.	40,210 household‐level observations (1 observational study)	⊕⊝⊝⊝ **Very low**^a^	
**Mean expenditure on taxed sugar‐added foods** Assessed with percentage change Follow‐up: 16 months	There was an effect of the intervention on the mean expenditure of taxed sugar‐added foods. Data show that the mean expenditure decreased after the implementation of the intervention slightly by 0.6% (SMD −0.006, 95% CI −0.03 to 0.02). The effect is based on very low‐certainty evidence.	40,210 household‐level observations (1 observational study)	⊕⊝⊝⊝ **Very low**^a^	
**Mean expenditure on untaxed sugar‐added foods** Assessed with percentage change Follow‐up: 16 months	The mean expenditure on untaxed sugar‐added foods increased after the implementation of the intervention by 3.0% (SMD 0.03, 95% CI −0.01 to 0.07). The effect is based on very low‐certainty evidence.	40,210 household‐level observations (1 observational study)	⊕⊝⊝⊝ **Very low**^a^	
**Difference in mean expenditure on taxed sugar‐added foods compared with untaxed sugar‐added foods** Assessed with percentage change Follow‐up: 16 months	The mean expenditure on taxed sugar‐added foods differed from the mean expenditure on untaxed sugar‐added foods after the implementation of the intervention by 3.7% (SMD −0.037, 95% CI −0.08 to 0.01). The effect is based on very low‐certainty evidence.	40,210 household‐level observations (1 observational study)	⊕⊝⊝⊝ **Very low**^a^	
**CI:** confidence interval; **SMD:** standardised mean difference				
**GRADE Working Group grades of evidence** **High certainty:** We are very confident that the true effect lies close to that of the estimate of the effect **Moderate certainty:** We are moderately confident in the effect estimate: The true effect is likely to be close to the estimate of the effect, but there is a possibility that it is substantially different **Low certainty:** Our confidence in the effect estimate is limited: The true effect may be substantially different from the estimate of the effect **Very low certainty:** We have very little confidence in the effect estimate: The true effect is likely to be substantially different from the estimate of effect				

^a^Non‐randomised study (downgraded to low), downgraded one additional level (to very low) for risk of bias due to simultaneous intervention of other taxes and likely misclassification of food products as to whether taxed or untaxed. Certainty is also affected by indirectness because the Hungarian tax is related to specific sugar contents in the particular food categories that were taxed, and the study measured purchased quantities and not consumption. Thus, it is not a direct representation of the effect of a complete tax on sugar or sugar‐added foods. *Effects are presented as SMDs as the number of distinct households and participants was not available to calculate valid MDs.

## Background

### Description of the condition

#### Epidemiological background

Preventive action comprising both policies and interventions are urgently needed to curb the obesity and overweight epidemics and their detrimental health impacts (WHO 2000). The World Health Organization (WHO) Commission on the Social Determinants of Health called for intersectoral action to address the social determinants of health to improve population health and health equity, including fiscal interventions such as taxes (CSDH 2008). The WHO Commission on Ending Childhood Obesity (ECHO) has highlighted the need for identifying and implementing effective policies and interventions that can curb overweight and obesity specifically among children (WHO 2016). In a fact sheet published in 2017, WHO argued the case for taxing sugary foods, specifically sugary drinks, to fight the obesity and overweight epidemics (WHO 2017). Overweight and obesity pose serious threats to global public health, with prevalences increasing over time in low‐, middle‐, and high‐income countries (De Onis 2010; James 2004; WHO 2000). According to the 2018 report from the World Health Organization (WHO 2018), based on data from 2016, the global prevalences of overweight (defined as a body mass index (BMI) of 25 or higher) are 39% for the total population with 39% for men and 40% for women, and of obesity (BMI of 30 or higher) are 13% of the total population with 11% for men and 15% for women (WHO 2018). In 2016, about 41 million children aged under five years were estimated to be overweight (WHO 2018). In some African countries, the prevalences of overweight and obesity are comparatively low at an estimated 16% and 3%, respectively, whereas in the Pacific Island countries and territories, prevalences for overweight and obesity are alarming, at up to 81% and 51%, respectively (WHO 2014). Moreover, the prevalence of overweight and obesity is growing rapidly with the highest prevalence seen in the American Regions (29%), the European Regions (23%) and the Eastern Mediterranean Regions (21%) (WHO 2018). About 7% of the population in low‐income countries is obese as compared to 25% of the population in high‐income countries (WHO 2018). Overweight and obesity are major risk factors for morbidity and mortality, with an attributable annual burden of about 3.4 million deaths and 93.6 million disability‐adjusted life years (DALYs) globally (WHO 2014). From a global perspective, the fatal and non‐fatal health loss that can be attributed to overweight and obesity is generally lower in middle‐ and high‐income countries than in low‐income countries (Dinsa 2012; Drewnowski 2004; Ng 2014; Robroek 2013; Salois 2012; Valera 2015; WHO 2009).

#### Social inequalities

As the WHO Commission on Social Determinants of Health and similar subsequent reports noted (CSDH 2008; Marmot 2012), the unequal distribution of overweight and obesity within and between countries also poses a serious challenge for achieving health equity nationally and globally. Within a country, overweight and obesity are usually (but not always) distributed along a social gradient. Regarding socioeconomic status, for example, higher prevalences of overweight and obesity are generally observed in people with a lower socioeconomic status. However, in some low‐income countries, such as Cameroon and many Pacific Island countries and territories, people with a higher socioeconomic status are relatively more likely to be overweight or obese. In some low‐ and middle‐income countries (e.g. China), the relationship of socioeconomic status with overweight and obesity, respectively, is unclear (Dinsa 2012; McLaren 2007; Ogden 2015; Wang 2012). Furthermore, it is possible that disadvantaged population groups may be at greater risk of obesity‐related harms, even without experiencing greater exposure levels (Diderichsen 2019).

#### Nutritional transitions

Across the globe, major dietary shifts are occurring, resulting in nutritional transitions. Nutritional transitions ‐ reflecting changes in diet, physical activity and health ‐ are major contributors to overweight and obesity becoming increasingly prevalent globally. In the last four decades, the daily food consumption rose by a global average of about 400 calories. However, the main sources of calorie intake greatly differ between low‐ and middle‐income countries and high‐income countries. In low‐ and middle‐income countries, calorie consumption increased between 1963 and 2003 for sugar (by 127%), meat (by 119%), and vegetable oils (by 199%), while in industrial countries, only consumption of vegetable oils increased substantially (by 105%) (Kearney 2010; World Bank 2015). In China – a major developing country that was classified as an upper‐middle income country by the World Bank – dramatic nutritional transitions have occurred over the past four decades, resulting in substantial increases in consumption of sugar (by 305%), meat (by 349%), and vegetable oils (by 680%) (Kearney 2010; World Bank 2015). However, in a country such as China, these changes occur more rapidly among people with lower incomes (Popkin 2002). Consumption of sugar notably increased in developing countries with lower incomes, particularly in Asia, Latin America and Africa. In high‐income countries, time trends of sugar consumption show regional differences: thus, some industrialised, high‐income regions, such as North America, show declines in sugar intake, whereas in others, such as Europe, consumption of sugar increased modestly (Kearney 2010). The prevalences of obesity and overweight are substantially higher among some indigenous populations than among non‐indigenous populations (Lee 1994). A major cause might be relatively higher consumption of sugar, sugar‐sweetened beverages (SSBs), and white flour among some indigenous populations, compared with non‐indigenous populations (Lee 1994). However, across the globe, Indigenous Peoples have undergone a relatively steeper and faster nutritional transition in recent decades, away from consumption of traditional foods to less healthier non‐traditional foods that are high in sugar, fat and carbohydrates, and more vitamins, proteins, zinc and magnesium (Kuhnlein 2004). The Third Strategic Report of the Mediterranean Diet Surveillance System noted that European Mediterranean countries underwent a ‘westernisation’ of nutritional patterns: consumption of vegetables declined, and intake of sugar, sweeteners, oil, and meat increased (Vareiro 2009). In contrast, Northern European countries transitioned into healthier nutritional patterns (Vareiro 2009).

#### Definition of 'unprocessed sugar' and 'sugar‐added foods'

Consumption of unprocessed sugars and sugar‐added foods contribute substantially to overweight and obesity (WHO 2018). We define 'unprocessed sugar' for the purpose of this review ‐ on the basis of the definitions of 'sugars' and 'free sugars' given below‐ as monosaccharides (such as glucose, fructose, and galactose), disaccharides (such as lactose, maltose, and sucrose) and higher saccharides (such as cellulose).

Traditionally, the term 'sugars' describes mono‐ and disaccharides (FAO/WHO 1998). Monosaccharides include fructose, galactose, and glucose. Disaccharides include lactose, maltose, sucrose, and trehalose. Some sweeteners, such as corn syrups, also consist of higher saccharides. In 2002, the Joint WHO and Food and Agriculture Organization of the United Nations (FAO) Expert Consultation introduced the term 'free sugars' (Amine 2002). In 2015, the definition of the term was elaborated for the WHO guideline on sugar intake for adults and children. 'Free sugars' are defined as mono‐ and disaccharides (such as lactose, maltose, and sucrose) that are added to foods (WHO 2015a).

We define 'sugar‐added foods' for the purpose of this review ‐ on the basis of the following definitions ‐ as non‐liquid food products (i.e. this review does not include drinks, including SSBs) that contain artificially‐added sugar in various quantities, where sugar refers to monosaccharides, disaccharides, and higher saccharides (as defined above).

Based on the definition of the US Department of Agriculture (USDA), added sugars are either pure sugars or natural products with high sugar content (e.g. honey) that are added to food during processing or preparation. In the preparation of a food product, sugars can be processed in any way, e.g. baked or cooked. Added sugar mainly appears in cakes, cookies, desserts, pies, and candy. "Specifically, added sugars include white sugar, brown sugar, raw sugar, corn syrup, corn‐syrup solids, high‐fructose corn syrup, maple syrup, pancake syrup, fructose sweetener, liquid fructose, honey, molasses, anhydrous dextrose, and crystal dextrose. Added sugars do not include naturally occurring sugars such as lactose in milk or fructose in fruits" (USDA/HHS 2000).

#### Effects of sugar consumption on health, society and economy

Overweight and obesity are risk factors for several diseases. Overweight and obesity are defined as an excess of adipose tissue in one’s body caused by an imbalance of energy intake and energy expenditure resulting from diverse genetic, environmental, cultural, behavioral, social and/or economic factors (Kopelman 2007; WHO 2015b). Increased energy intake is the result of overconsumption of foods and especially consumption of surplus quantities of high‐caloric foods. Unprocessed sugar and sugar‐added foods are a main source of excessive calorie intake (Bowman 2004; Popkin 2003). Thus, a sugar‐rich diet, especially when combined with physical inactivity, may cause overweight and obesity, which, in turn, increases the risk of high blood pressure (e.g. hypertension), dyslipidaemia, peripheral insulin resistance, inflammation, and dental caries (Kopelman 2007; Moynihan 2014; WHO 2015b). These adverse effects of overweight and obesity may lead to substantial health loss across many bodily systems, including disorders of the cardiovascular (e.g. ischaemic heart disease), gastrointestinal (e.g. bowel cancer), musculoskeletal (e.g. osteoarthritis), endocrine (e.g. type 2 diabetes mellitus), and respiratory (e.g. obstructive sleep apnoea) systems (Aronne 2002). In addition to its contribution to specific diseases, obesity may also reduce psychological well‐being at the individual level and adversely affect societies and economies at the population level by, for example, reducing economic productivity and increasing demands on healthcare resources (Colditz 1999; Wardle 2005). Overweight and obesity in childhood and adolescence are associated with increased risks of overweight and obesity in adulthood (Power 1997). Thus, early development of overweight and obesity has substantial and long‐lasting consequences for a person's physical and mental health (Must 1999; WHO 2016).

Overweight and obesity are the most often cited effects of a sugar‐rich diet. However, the effects of a sugar‐rich diet are far‐reaching. For instance, in the USA, dental caries is one of the most prominent childhood diseases with a minimum of one filing or caries lesion among 77.1% of children aged 0 to 17 years (Touger‐Decker 2003). Worldwide, one in 10 people is affected by diabetes (Basu 2013; James 2018).

Different anthropometric measures are used to evaluate overweight and obesity, including body weight, BMI, skinfold thickness, bone‐mineral density, waist circumference (WC), waist‐to‐hip ratio (WHR), and waist‐to‐height ratio (WHtR). Useful measures are also derived from more advanced measurement tools, such as bioelectrical impedance analysis (BIA), magnetic resonance imaging (MRI), isotope dilution analysis (IDA), ultrasound and computed tomography (CT) (WHO 2000).

Overweight and obesity incur direct costs (e.g. disease‐related preventive, treatment and diagnosis service costs) and indirect costs (e.g. disease‐related costs of lost productivity), both in the health sector and in other sectors, including labour and economic development (Van Nuys 2014; Wolf 1998). A systematic review on the direct costs of obesity estimated that it accounts on average for 0.7% to 2.8% of a country’s total healthcare expenditure (Withrow 2011). In the USA, treating overweight and obesity consumes 5% to 10% of the total healthcare costs, an estimated USD 120.1 to 240.2 billion in absolute terms (Tsai 2011). Indirect costs of overweight and obesity are higher than direct costs, accounting for 54% to 59% of the total cost estimates (Dee 2014). Moreover, according to a systematic review, overweight and obesity cause wage losses, especially among white women in the USA: a weight increase of 2 standard deviations (about 64 pounds) from the average weight was associated with a 9% lower wage (Cawley 2004).

### Description of the intervention

#### Food–related fiscal policies

Food‐related fiscal policies generally aim to either lower prices (e.g. subsidisation) or increase prices (e.g. taxation) for specific food groups. We evaluated the effects of taxes on unprocessed sugar and sugar‐added foods (as defined above). The Organization for Economic Co‐operation and Development (OECD) defines taxes as "compulsory unrequited payments to general government" (OECD 2014).

##### Typologies of taxes

There are two different types of indirect taxes with subcategories on sugar‐related products as shown in Figure 1: (1) import (or export) taxes (or fees) on unprocessed sugar or sugar‐added foods, and (2) within‐border (local, regional, national, and international) taxes (Fletcher 2010; Meessen 2007; Mytton 2012). From the perspective of the WHO Commission on Social Determinants of Health and its recommendations for actions (CSDH 2008), food‐related fiscal policies can be classified as an intersectoral socioeconomic intervention on the social determinants of health to improve health equity (Pega 2017a).

**1:**
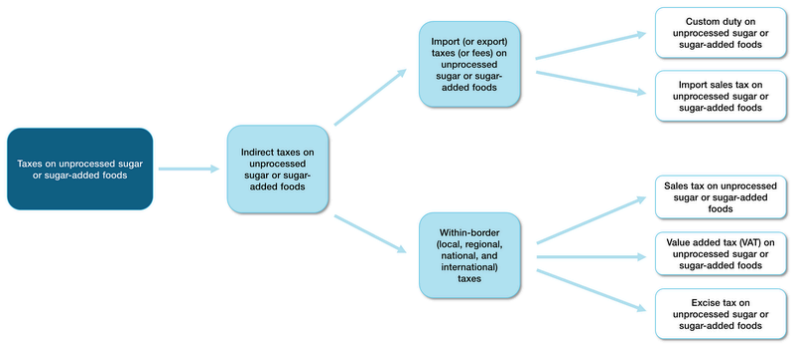
Typologies of taxes on unprocessed sugar or sugar‐added foods

###### Description of types of food‐related taxes

Indirect taxes are paid by the consumer, collected by the seller or intermediary, and forwarded to government. Sales taxes – as one form of indirect taxes ‐ are paid by the consumer at the moment of purchase of the taxed goods and services. Sales taxes are common tax interventions to reduce the consumption of a specific good, such as unprocessed sugar or sugar‐added foods (Brownell 2009). Value Added Tax (VAT) is the most popular tax across the globe and the principal type of indirect taxes. The term 'VAT' is used as a synonym for 'goods and services tax'. The underlying principle of the VAT system includes "the application to goods and services of a general tax on consumption exactly proportional to the price of the goods and services" (Schenk 2015). VAT is more commonly applied to different food categories than are targeted food taxes (Mytton 2007). The level of a sales tax may differ according to the type of to‐be‐taxed product and service. Sales taxes and VAT are added to the price of an item without consideration of the item's volume. Thus, goods of a larger size, that in most cases are comparably cheaper than the same goods of smaller sizes, result in a lower impact of the tax in goods with larger package sizes. An excise tax is an inland tax on the (production for) sale and the goods produced for sale. Custom duties (or 'border taxes') are taxes applied to imported products. The Cook Islands and Fiji, for example, implemented such custom duties on SSBs to increase the cost of these drinks as a means to fight the obesity epidemic (Snowdon 2013). Governments similarly also use import sales taxes, these being taxes on goods imported from countries that are not a contracting party of the importing country (Cnossen 1993). All taxes may encourage a reformulation of the taxed item to lower its price and thus, decrease the content of the taxed ingredient in a processed food product. For an overview on the different tax typologies, as described above, see Figure 1.

##### Aims and rationales of food‐related taxes

Fiscal policies such as excise taxes on food have been proposed, developed and implemented, generally with the goal of curbing overweight and obesity, but sometimes also to increase governmental revenue (Kim 2006). Taxes raise revenue for government, and these revenues may or may not be earmarked (also referred to as hypothecated) for public health programmes. These types of food taxation policies include taxes on salt, fats, SSBs, and unprocessed sugar or sugar‐added foods (other than beverages) more generally.

The underlying policy and economic rationale for implementing food taxation policies, including those on unprocessed sugar and sugar‐added foods, is a government’s motivation to create or increase a financial charge for a specific (unhealthy) food in order to increase consumer prices and usually also to raise public revenue. This price increase may then lead to a decrease in demand, which in turn may reduce the intake of the taxed (unhealthy) food product by reducing its consumption in the population (Ecorys 2014).

The implementation of food taxes may lead to changes in food composition, in an effort to minimise taxes paid. In other words, in response to the implementation of a tax on unprocessed sugar or sugar‐added foods, food industries may reformulate their products (Brownell 2009). This may lead to products with lower added sugar content, with potential benefits to human health. However, on the other hand, this reformulation of the product may make it even unhealthier, e.g. by adding other ingredients, such as fat, with potential detrimental health effects.

##### Focus of this review

This review will focus specifically on the taxation of unprocessed sugar and foods that contain added sugar (e.g. sweets, ice cream, confectionery, and bakery products) regardless of the taxation level. In tandem, we are also conducting systematic reviews of the effectiveness of taxes of fats (Lhachimi 2016), and SSBs (Heise 2016), for improving human health.

### How the intervention might work

See Figure 2 for a logic model describing the causal pathways through which taxation of unprocessed sugar and sugar‐added foods may work to reduce overweight, obesity and other health outcomes.

**2:**
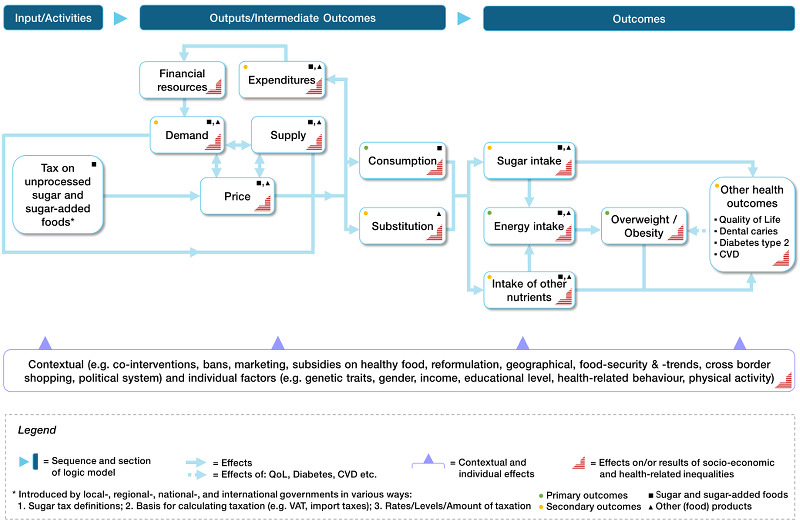
Study's initial logic model with causal pathways

The typical aim of prevention and treatment of overweight and obesity is weight reduction. This can be achieved by decreasing energy intake through changes in dietary behaviours (e.g. reduce consumption of foods high in added sugar and fats), drug treatment, a surgical intervention, or increased energy expenditure through increased physical activity, or a combination of some or all of these (Wadden 2002). Taxation of food might be an effective mechanism in reducing overweight and obesity prevalence.

In general, food taxes are often hypothesised to lead to reduced consumption of unhealthy foods (Mytton 2012). However, the decrease from food taxes in the percentage share of unprocessed sugar and sugar‐added food consumption in the total energy intake is likely to have one of two effects on health‐related behaviour: either it may lead to a reduction in total daily energy intake, or the consumption of unprocessed sugar and sugar‐added foods may be substituted by foods that are also relatively high in calories (e.g. high fat content) or by other unhealthy products, such as cigarettes and salts (Briggs 2013). While the former may lead to weight reduction, the latter may result in (1) weight gain, (2) a zero effect, or (3) weight reduction (Ecorys 2014). In any case, any effects of food taxation on public health and consumption patterns either take some time to become detectable (Fletcher 2010; Meessen 2007) or only show short‐term effects (Wansink 2014).

According to economic theory, the taxation of unprocessed sugar or sugar‐added foods is expected to cause an increase in price, which in turn will lead to a decrease in demand, sales, and consumption (Mytton 2012). Across cultures, a higher product price is also associated with a higher product reputation and quality (Dawar 1994). Thus, as a response to a tax implementation, the consumption of taxed items may rise. With regard to within‐country inequalities, as the price of a product determines the level of affordability, low‐income groups are usually more strongly affected by taxation policies than higher‐income groups (Eyles 2012; Maniadakis 2013). If low‐income populations have higher prevalences of overweight, obesity, type 2 diabetes, dental caries and other sugar‐related diseases and conditions than middle‐ and high‐income populations, then unprocessed sugar or sugar‐added foods taxation policies may disproportionately reduce consumption of unprocessed sugar and sugar‐added foods among the low‐income population, and thus improve health equity in the population. Furthermore, with regard to between‐country inequalities, these tax interventions may reduce overweight, obesity, type 2 diabetes, dental caries and other sugar‐related diseases and conditions differently across countries of different income levels. For example, it is theoretically plausible that such taxes are more effective in reducing sugar‐related diseases and conditions in low‐income countries than in middle‐ and high‐income countries. Thus, taxes on unprocessed sugar and sugar‐added foods have the potential to also improve between‐country health equity (Eyles 2012; Lorenc 2012; Maniadakis 2013).

In several countries, food taxes were implemented in the past and some were subsequently abolished. Table 3 gives an overview of the implemented and abolished food taxes worldwide based on information from countries' governmental websites and the NOURISHING framework of the World Cancer Research Fund International and (World Cancer Research Fund International 2019). Most of the food taxes implemented across countries are taxes on SSBs. However, some countries implemented taxes on unprocessed sugar and sugar‐added foods. For example, Norway taxes unprocessed sugar, sugar products and chocolate (Ecorys 2014; Norwegian Ministry of Finance 2015); Finland has taxed ice cream and confectionery (tax abolition in January 2017; Ecorys 2014); Hungary taxes pre‐packaged foods high in added‐sugar content (i.e. chocolates, sweets, biscuits and ice creams; Ecorys 2014; Holt 2011); Denmark temporarily taxed ice cream, chocolate and confectionery (Wilkins 2010); Bermuda taxes sugar confectionery, chocolate and other foods containing cocoa and sugar; Dominica taxes sweets, candies and chocolate bars; India taxes all goods containing added sugars; St. Vincent and the Grenadines tax brown sugar; and the Navajo Nation (USA) taxes pre‐packaged and non‐pre‐packaged snacks high in sugar including sweets and crisps (World Cancer Research Fund International 2019).

**1:** Food taxes worldwide

**Country**	**Tax implementation**	**Tax abolition**	**Taxed items**	**Tax rate**	**Exempted from tax**
Bahrain	30 December 2017	‐	Energy drinks	100%	‐
			SSBs	50%	‐
Barbados	1 August 2015	‐	SSBs	10%	100% natural fruit juices, coconut water, plain milk, evaporated milk
Belgium	26 December 2015	‐	SSBs	0.03 EUR/L (around USD 0.03)	
	1 January 2016	‐	SSBs	0.068 EUR/L (excise duty, around USD 0.076)	‐
			Liquid for manufacturing SSBs	0.41 EUR/L (around USD 0.46)	‐
			Powder for manufacturing SSBs	0.68 EUR/kg (around USD 0.76)	‐
**Bermuda**	**1 October 2018**	‐	SSBs/mineral waters and aerated waters containing other sweetening matter or flavouring	50%	‐
			Syrups containing sugar or other sweetening matter	50%	Except fruit/vegetable juices
			**Sugar confectionery, not containing cocoa**	**50%**	‐
	**1 April 2019**	‐	**Sugar confectionery (including white chocolate), not containing cocoa**	**75%**	‐
			**Chocolate, other food preparations containing cocoa and added sugar**	**75%**	‐
			Preparations for making beverages, containing added sugar	75%	‐
			Mineral/aerated waters, containing added sugar	75%	‐
Brunei	1 April 2017	‐	SSBs, containing > 6 g of total sugar/100 mL	0.40 BND/L (around USD 0.28)	Milk‐based beverages and fruit juices
			Soya milk drinks, containing > 7 g of total sugar/100 mL		
			Malted or chocolate drinks, containing > 8 g of total sugar/100 mL		
			Coffee‐based/flavoured drinks containing 6 g of total sugar/100 mL		
Chile	1 October 2014	‐	SSBs and energy drinks, containing > 6.25 g of sugar/100 mL	13%	
	1 January 2015	‐	SSBs and energy drinks, containing > 6.25 g of sugar/100 mL	18%	‐
			Sugary drinks, containing < 6.25 g of sugar/100 mL	10%	‐
Denmark	1 October 2011	1 January 2013	Products exceeding 2.3 g saturated fat per 100 g fat (e.g. including meat, animal fat, dairy products, margarine, spreads, edible vegetable oils and fats)	16 DKK/kg (around USD 2.70)	‐
**Dominica**	**1 September 2015**	‐	**Sweets, candy, chocolate bars**	**10%**	‐
			SSBs and energy drinks high in sugar		
Ecuador	May 2016	‐	SSBs, containing < 25 g sugar/L	10%	Dairy products + derivatives, mineral water, juices with 50% natural content
			All energy drinks	10%	
			SSBs > 25 g sugar/L	USD 0.0018/g (of sugar)	
Fiji	2007	‐	Locally produced SSBs	10 cents/L (around USD 0.05/L)	‐
			Imported SSBs	10%	‐
			Imported powders, preparations for manufacturing beverages, flavoured and coloured sugar syrups	10%	‐
	2011	‐	Imported SSBs	15%	‐
	23 June 2016	‐	Locally produced SSBs	30 cents/L (around USD 0.15)	‐
	August 2017	‐	Locally produced SSBs	35 cents/L (around USD 0.17)	‐
			Imported powders, preparations for manufacturing beverages, flavoured and coloured sugar syrups	10%	
**Finland**	2014	‐	SSBs, containing >0.5% sugar	0.22 EUR/L (around USD 0.24/L)	producers with an annual production volume of <50,000 litre
			SSBs, containing ≤0.5% sugar	0.11 EUR/L (around USD 0.12/L)	
	**2014**	**1 January 2017**	**Confectionery, ice cream**	**0.95 EUR/kg (around USD 1.06**/L**)**	‐
France	1 July 2018	‐	SSBs	Tax is proportional to the sugar content, i.e. 0.135 EUR (around USD 0.15) for 10 g added sugar/L	‐
			Non‐calorically SSBs	3 EUR/hL (around USD 3.34)	‐
French Polynesia	2002	‐	Domestically produced SSBs	40 XPF/L (around USD 0.44)	‐
			Imported SSBs	60 XPF/L (around USD 0.68)	
**Hungary**	see Table 4				
**India**	**1 July 2017**	‐	**All goods (including aerated waters), containing added sugar, other sweeteners, flavourings**	**28% + cess 12%**	‐
Ireland	1 May 2018	‐	SSBs, containing 5 g‐8 g sugar/100 mL	20 cents/L (around USD 0.23)	Fruit juices, dairy products
			SSBs, containing > 8 g sugar/100 mL	30 cents/L (around USD 0.35)	
Kiribati	2014	‐	SSBs, containing added sugar, other sweeteners, flavourings	40%	Fruit/vegetable juices, fruit concentrates
Latvia	1 May 2004	‐	SSBs, containing added sugar, other sweeteners, flavourings	2.85 EUR/100 L (around USD 3.17)	Fruit/vegetable juices, nectar, beverages containing > 90% juice (not made out of fruit concentrate), < 10% added sugar, not containing food additives/flavourings, natural/mineral water, water enriched with minerals/vitamins, not containing added sugar/sweeteners/flavourings
	2016	‐	SSBs, containing added sugar/other sweeteners/flavourings	7.40 EUR/100litre (around USD 8.23)	
Mauritius	October 2016	‐	SSBs (including juices, milk‐based beverages, soft drinks)	0.03 MUR/g of sugar (around USD 0.0008)	‐
Mexico	1 January 2011	‐	Energy drinks (non‐alcoholic beverages containing > 20 mg/100 mL of caffeine and mixed stimulants)	25%	‐
			Concentrates, powders, syrups for manufacturing energy drinks		
	1 January 2014	‐	SSBs	1 MXN/L (around USD 0.05)	milks, yoghurts
			Food with high caloric density containing ≥ 275 calories/100 g (including chips, snacks, confectionery, chocolate, cacao‐based products, puddings, peanut butter, hazelnut butter)	8%	‐
Morocco	1 January 2019	‐	Manufactured or imported SSBs	50%	‐
			Carbonated or non‐carbonated (mineral/table) water or others containing < 10% of edible fruit juice or juice concentrates	MAD 0.45/L (about USD 0.04)	‐
			Carbonated or non‐carbonated (mineral/table) water or others containing > 10% fruit juice or juice concentrates	MAD 0.15/L (about USD 0.016)	‐
			Lemonades containing sugar and < 6% lemon juice or concentrate equivalent	MAD 0.45/L (about USD 0.04)	‐
			Lemonades containing sugar and > 6% lemon juice or concentrate equivalent	MAD 0.15/L (about USD 0.016)	‐
			Unfermented (non‐)carbonated beverages containing malt extracts/natural fruit flavourings, sweetened with sucrose, dextrose, glucose, fructose, maltose or a mixture	MAD 1.24/L (about USD 0.13)	‐
			Energy drinks containing ≥ 2 stimulant ingredients e.g. caffeine, taurine, glucuronolactone	MAD 6.00/L (about USD 0.62)	‐
**Norway**	**2017**	‐	SSBs	3.34 NOK/L (around USD 0.40)	‐
			Concentrated syrups	20.32 NOK/L (around USD 2.44)	
			**Chocolate**	**20.19 NOK/kg (around USD 2.43)**	
			**Sugar products**		
			**Sugar**	**7.81 NOK/kg (around USD 0.94)**	
	**January 2018**	‐	**Chocolate**	**36.92 NOK/kg (around USD 4.69)**	‐
			**Sugar products**		‐
Palau	September 2013	‐	SSBs	USD 0.28175/L	‐
Peru	10 May 2018	‐	SSBs, containing ≥ 6 g sugar/100 mL	25%	Beverages < 6 g sugar/100 mL
Portugal	1 February 2017	‐	SSBs, containing < 80 g sugar/L	0.08 EUR/L (around USD 0.10)	‐
			SSBs, containing > 80 g sugar/L	0.16 EUR/L (around USD 0.20)	
Qatar	01 January 2019	‐	SSBs		Carbonated non‐flavoured waters, coffee, tea
			SSB concentrates, powders, gels, extracts	50%	
			Energy drinks, containing stimulant substances (e.g. caffeine, taurine, ginseng, guarana)	100%	
Samoa	1984	‐	SSBs	0.3 Samoan Tala/L (around USD 0.12)	‐
	2008	‐	SSBs	0.4 Samoan Tala/L (around USD 0.17)	‐
	2012	‐	High‐fat turkey tail	300% (import duty)	
	2014		High‐fat turkey tail	100% (import duty)	‐
Saudi Arabia	9 June 2017	‐	Energy drinks	100%	Differences in rates depending on the nature of the product ‐ user manual
			SSBs	50%	
South Africa	December 2017	‐	SSBs, containing > 4 g sugar/L	2.1 cents/g of sugar (around USD 0.17)	Fruit/vegetable juices
Spain (Catalonia)	1 May 2017	‐	SSBs, containing 5 g‐8 g sugar/100 mL	0.08 EUR/L (around USD 0.09)	Natural fruit juices, alcoholic beverages, sugar‐free soft drinks, alternatives to milk with no added caloric sweeteners
			SSBs, containing > 8 g sugar/100 mL	0.12 EUR/L (around USD 0.13)	
St. Helena	27 May 2014	‐	SSBs, containing ≥ 15 g sugar/L	0.75 St Helenian pound/L (around USD 0.95)	‐
**St. Vincent and the Grenadines**	**1 May 2016**	‐	**Brown sugar**	**15%**	‐
Thailand	16 September 2017	‐	Artificial mineral water, soda water, carbonated soft drinks without sugar or other sweeteners and without flavour; mineral water, carbonated soft drinks with added sugar or other sweeteners of flavours	14%	‐
			Fruit and vegetable juices	10%	
			SSBs, containing 6 g‐8 g sugar/L	10%/14% + 0.10 THB/L (around USD 0.0031)	
			SSBs, containing 8 g‐10 g sugar/L	10%/14% + 0.30 THB/L (around USD 0.0095)	
			SSBs, containing 10 g‐14 g sugar/L	10%/14% + 0.50 THB/L (around USD 0.015)	
			SSBs, containing >14 g sugar/100 mL	10%/14% + 1 THB/L (around USD 0.031)	
Tonga	2013	‐	SSBs	1 TOP/L (around USD 0.50)	‐
			Animal fat products	1 TOP/kg (around USD 0.45)	
			Turkey tails	1 TOP/kg (around USD 0.45)	
	2016	‐	Animal fat products	2 TOP/kg (around USD 0.90)	
			Turkey tails	1.5 TOP/kg (around USD 0.70)	
UAE	1 October 2017	‐	SSBs	50%	Unflavoured aerated water
			SSB concentrations, powders, gel, extracts	50%	‐
			Energy drinks, containing stimulant substances	100%	‐
UK	April 2018	‐	SSBs, containing ≥ 5 g and < 8 g of sugar/100 mL	0.18 GBP/L (around USD 0.25)	Milk‐based or substitute drinks, pure fruit juices, any other drinks with no added sugar, alcohol substitute drinks, soft drinks of a specified description for use for medicinal or other specified purposes
			SSBs, containing ≥ 8 g of sugar/100 mL	0.24 GBP/L (around USD 0.34)	
USA: California, Berkeley	March 2015	‐	SSBs	USD 0.01/ounce (equivalent 28.33 g) of sugar sweetened beverages	Infant formula, milk products, natural fruit and vegetable juices
USA: California, Oakland	1 July 2017	‐	SSBs, containing ≥ 1 caloric sweetener or ≥ 25 calories/12 fluid ounces (equivalent 354.84 mL) of beverage	USD 0.01/ounce (equivalent 28.33 g)	Milk products, 100% juice, infant or baby formula, diet drinks, drinks taken for medical reasons
USA: California, San Francisco	1 January 2018	‐	SSBs, containing added sugar and > 25 calories/12 ounces (equivalent 339,96 g)	USD 0.01/ounce (equivalent 28.33 g)	Beverages containing solely 100% juice, artificially sweetened beverages, infant formula, milk products
			SSB syrups/powders		
USA: City of Albany	1 April 2017	‐	SSBs	USD 0.01/ounce (equivalent 28.33 g)	Infant formula/milk products/ natural fruit/vegetable juices
USA: Colorado	1 July 2017	‐	SSBs, containing ≥ 5 g of caloric sweetener/12 fluid ounces (equivalent 354.84 mL)	USD 0.02/ounce (equivalent 28.33 g)	Milk products, infant formula, alcoholic beverages, beverages for medical use, distribution of syrups and powders sold directly to a consumer intended for personal use
**USA: Navajo Nation**	**1 April 2015**	‐	**Minimal‐to‐no‐nutritional value food items (including SSBs, pre‐packaged and non‐pre‐packaged snacks stripped of essential nutrients and high in salt, saturated fat and sugar including sweets, crisps and chips)**	**2%**	‐
USA: Pennsylvania	1 January 2017	‐	SSBs	USD 0.15/ounce (equivalent 28.33 g)	‐
			Any non‐alcoholic syrups, other concentrate used in beverages	USD 0.15/ounce (equivalent 28.33 g) on the resulting beverage	
USA: Washington	1 January 2018	‐	SSBs	USD 0.175/fluid ounce (equivalent 29.57 mL)	Beverages containing < 40 calories/12 ounces (equivalent 339,96 g), including beverages with milk as the principal ingredient, 100% natural fruit and vegetable juice, meal replacement beverages, infant formula, concentrates used in combination with other ingredients to create a beverage
			SSBs from manufacturers (worldwide income of > USD 2 million and < USD 5 millions)	USD 0.01/ounce (equivalent 28.33 g)	
Vanuatu	9 February 2015	‐	SSBs	50 VUV/L (around USD 0.47)	‐
**BND:** Brunei Dollar; **DKK:** Danish Kroner; **GBP:** Great Britain Pound Sterling; **MAD:** Morrocan Dirham; **MUR:** Mauritius Rupee; **MXN:** Mexican Peso; **NOK:** Norwegian Krone; **SSB:** sugar‐sweetened beverage; **THB:** Thai Baht; **TOP:** Tonga Pa'anga; **USD:** United States Dollar; **VUV:** Vanuato Vatu; **XPF:** CFP Franc					

Information is derived from countries' governmental websites and World Cancer Research Fund International 2019.

How the taxation of unprocessed sugar and sugar‐added foods might affect outcomes is described in a logic model with causal pathways (Figure 2). The taxation of unprocessed sugar and sugar‐added foods ‐ introduced by local, regional, national, or multinational governments ‐ is hypothesised to result in price changes (e.g. increased prices of chocolate, ice cream, and bakery products; Epstein 2012; Jensen 2013; Maniadakis 2013), which in turn may lead to altered expenditure patterns for food. Financial resources ‐ also dependent on expenditure on food ‐ and contextual and individual factors (e.g. income), determine the demand for food products. These market components impact consumer purchases and consumption choices for different food categories, including unprocessed sugar and sugar‐added foods (Briggs 2013; Sharma 2014). This may result in a lower intake of the taxed food products (unprocessed sugar and sugar‐added foods) and in a substitution of these by other (food) products (Fowler 2015; Yang 2010). As a consequence, food tax‐induced changes in consumption patterns should result directly in changes to intake of unprocessed sugar and sugar‐added foods (Epstein 2012; Maniadakis 2013). A decrease in the intake of unprocessed sugar and sugar‐added foods ‐ as one hypothesised consequence of taxing these foods ‐ can reduce overweight, obesity, and other health outcomes, both directly and indirectly. To exemplify the direct path from the intake of unprocessed sugar and sugar‐added foods to other health outcomes, a decrease in the intake of unprocessed sugar and sugar‐added foods has the potential to reduce the risk of dental caries (Moynihan 2014; WHO 2015a). The indirect path from the intake of unprocessed sugar and sugar‐added foods to overweight and obesity operates through energy intake. For example, a decreased energy intake as a consequence of decreased intake of unprocessed sugar and sugar‐added foods lowers the risk of being overweight and obese, respectively (Kim 2006; Malik 2013). Moreover, food tax‐induced changes in consumption patterns may directly result in changes in nutrient intake (Epstein 2012; Maniadakis 2013). The direct path from intake of other nutrients (e.g. fat or dietary minerals) as a consequence of substitution effects has the potential to directly increase, decrease or not affect the risk of other health outcomes (e.g. fatty liver). The indirect path from intake of other nutrients to overweight, obesity and other health outcomes goes through energy intake. To illustrate this, a higher intake of other nutrients (e.g. saturated or unsaturated fat) as a substitution effect of decreased intake of unprocessed sugar and sugar‐added foods affects energy intake (increase, decrease or zero effect) and is therefore associated with the risk of overweight, obesity and other health outcomes (Marriott 2010). Decreased risks of overweight and obesity, in turn, can reduce the risk of developing other diet‐related diseases and conditions (e.g. chronic diseases such as type 2 diabetes, cardiovascular diseases, and dental caries; Guh 2009).

Contextual and individual factors (see Figure 2) influence the process from the input to the outcomes, alter effect sizes and help us to understand the causal relationships (Qi 2012). Alternative interventions may be possible comparators but also potential co‐interventions (i.e. complementary interventions to reduce the consumption of unprocessed sugar and sugar‐added foods, such as bans on marketing, which are designed to enhance intervention effectiveness). Therefore, the effect of taxation may be modified by other interventions by governments, communities and the food or other (e.g. agricultural) industry to reduce consumption of unprocessed sugar and sugar‐added foods (Jou 2012; Thow 2010; Thow 2011; Thow 2014). Social factors such as gender and educational attainment may determine the effectiveness of a tax intervention at the individual level, and tax interventions may thus impact individual health, population health and health equity (Anderson 2011b).

### Why it is important to do this review

There is increasing public health interest in taxing unprocessed sugar and sugar‐added foods as an intervention, sometimes spurred by the recent implementation of food taxes in several countries, such as Hungary and Norway. However, the implementation of a tax on unprocessed sugar and sugar‐added foods is only one of many policy options for reducing consumption of these foods (Hawkes 2015).

Consumption of unprocessed sugar and sugar‐added foods is far above recommended levels. In 2018/2019, world sugar consumption was 173.95 million metric tonnes (Statista 2019). Data from 2010 and 2011 suggest that the average daily per capita consumption of sugar is about 63 g. This differs by country, with the lowest intake being observed in Bangladesh (approximately 22 g) and the highest in Israel (approximately 181 g; Groupe Sucre et Denrées 2015).

WHO recommends a daily sugar consumption of less than 10% of the total energy intake. Thus, the recommended maximum level in adults is approximately 50 g. Keeping the daily sugar intake on a level below 5% (approximately 25 g) of the recommended total energy intake might have even greater health benefits (WHO 2015a). In view of the excess consumption of sugar and the worldwide increase in overweight and obesity prevalence, governments must urgently act. Taxes for unprocessed sugar and sugar‐added foods are interventions that may help to fulfil the policy aim of reducing the prevalence of overweight and obesity and the associated burden of disease, and the associated costs to the health and other sectors.

Previous systematic reviews have investigated relevant public health effects of taxing fast food (Powell 2013), SSBs (James 2018; Maniadakis 2013; Nakhimovsky 2016; Powell 2013; Redondo 2018; Teng 2019; Welsh 2013), and saturated fat (Eyles 2012; Maniadakis 2013; Powell 2013), and subsidies of fruits and vegetables (Eyles 2012; Powell 2013), or all foods (Green 2013; Niebylski 2015; Powell 2013). Some of these reviews have combined diverse fiscal policy interventions in assessing the association between food pricing strategies and relevant public health outcomes (Maniadakis 2013; Powell 2013; Welsh 2013). Results as to the effectiveness of fat taxes and food subsidies are inconsistent across systematic reviews, suggesting no effects (Maniadakis 2013; Powell 2009), or beneficial effects for relevant public health outcomes (Eyles 2012; Green 2013; Powell 2013). Inconsistency of results across systematic reviews may arise from the investigation of different policy interventions: the inclusion of studies of different (and non‐comparable) populations (e.g. populations defined by different socioeconomic status); and the inclusion of different study types (e.g. simulation studies only or cross‐sectional studies in combination with other study types).

This review is different from previous reviews that investigated the effectiveness of food taxes and subsidies for the improvement of population health and changes in consumption patterns (Eykelenboom 2019; Eyles 2012; Maniadakis 2013; Niebylski 2015; Powell 2009; Powell 2013; Teng 2019; von Philipsborn 2019). This is the first systematic review to investigate the effects of taxes of unprocessed sugar and non‐liquid sugar‐added foods. Evidence is required regarding the effectiveness of taxing unprocessed sugar and sugar‐added foods so that policy makers can make evidence‐based decisions.

This research is part of a series of three systematic reviews of different types of food taxation carried out by the same author group using a similar methodological approach. For reasons of comparability, the methodological content is similar across the three reviews. These reviews focus on the effects of governmental taxation to increase the prices of: (1) unprocessed sugar or sugar‐added foods (this review), (2) processed or packaged food with high content of saturated fat (Lhachimi 2016), and (3) SSBs (Heise 2016).

## Objectives

To assess the effects of taxation of unprocessed sugar or sugar‐added foods in the general population on the:

- consumption of unprocessed sugar or sugar‐added foods;
- prevalence and incidence of overweight and obesity; and
- prevalence and incidence of other diet‐related health outcomes.

## Methods

### Criteria for considering studies for this review

#### Types of studies

Our pre‐published review protocol guided this review (New Reference). We included various study designs and adopted an approach previously used in at least two other Cochrane Reviews in order to summarise ‘best available evidence’ (Gruen 2004; Turley 2013). This approach clearly separates studies into two broad categories: (1) studies meeting rigorous Cochrane Effective Practice and Organisation of Care (EPOC) criteria (EPOC 2012; EPOC 2015), and (2) supporting studies ‐ those not meeting EPOC criteria with greater risk of bias as well as lower external generalisability. First, for the synthesis of main results, in line with EPOC criteria we included:

- randomised controlled trials (RCTs);
- cluster‐randomised controlled trials (cRCTs);
- non‐randomised controlled trials (nRCTs);
- controlled before and after (CBA) studies; and
- interrupted time series (ITS) studies.

As recommended by EPOC, we included controlled studies with more than one intervention or control site and ITS studies with a clearly defined intervention time and at least three data points before and three after the intervention (EPOC 2012).

There were no restrictions by publication date and language, but we only included studies focusing on humans (CPH 2011). We had no restriction on study duration and participants. Closed field experiments suggest that consumer behaviour adaptations, expressed in terms of sales of unprocessed sugar or sugar‐added foods, become apparent within a short time frame, such as one month (Block 2010). Implementation of taxes on sugar or sugar‐added foods at a national level might feature a longer time lag between intervention and outcomes, especially for health outcomes. However, in one study the efficacy of food taxes with respect to purchases was apparent after one year (Popkin 2016). In general, field experiments on food taxes recruit small numbers of participants. Nevertheless, they were considered as a valuable source to identify important outcome pathways and effects on food patterns relevant to the taxation of unprocessed sugar or sugar‐added foods (Epstein 2012).

We excluded simulation studies due to their potential limitations from their underpinning assumptions (e.g. lack of potential supply‐side changes, static models to predict weight loss), and other methodological restrictions (e.g. the use of a combination of heterogeneous data sources; Lin 2011; Shemilt 2015).

##### Supporting studies

We considered as supporting studies:

- studies that use an RCT, cRCT, nRCT, CBA or ITS design but do not fulfil the EPOC criteria (hence, are not included in the main results as outlined above);
- prospective cohort studies;
- retrospective/non‐concurrent cohort studies;
- repeated cross‐sectional studies; and
- uncontrolled before‐after (UBA) studies.

However, we did not find any eligible supporting studies.

In future updates, we will stick to our initially planned methodology on supporting studies. We originally planned not to include 'supporting studies' in the statistical synthesis of the primary included studies (i.e. alongside those meeting EPOC criteria), but aimed to narratively synthesise them in addition to the main findings. We planned to extract the same type of data from supporting studies as we did for the included studies and planned to document these in a separate 'Characteristics of supporting studies' table. We planned to carry out 'Risk of bias' assessments on these studies and to undertake quality assessments using the GRADE approach, then to present the findings from these supporting studies separately, as supplemental information in the results section and in a separate 'Summary of findings' table. We planned to make observations as to similarities and differences of findings between the included studies and the supporting studies in the 'Discussion' section, to help summarise the breadth, quality and findings of the totality of research on the effects of these interventions.

In future updates, supporting studies may either support or challenge results in the main findings and highlight uncertainty and potential research gaps. We will consider known limitations of UBA studies, cohort studies, and repeated cross‐sectional studies, especially confounding or lack of control for underlying time trends, when we assess these studies' eligibility for inclusion. If UBA studies, cohort studies, and repeated cross‐sectional studies are likely to be biased and do not use analytic strategies (e.g. stratification) or other designs (e.g. regression discontinuity (Craig 2017), fixed effects regression (Gunasekara 2014) or marginal structural models (Pega 2016)), to control for confounders and time trends, we will exclude these studies from the 'supporting studies' analysis.

#### Types of participants

We included studies of children (0 to 17 years) and adults (18 years and over) from any country and setting.

We excluded studies investigating the effects of taxing unprocessed sugar or sugar‐added foods focusing on specific subgroups, particularly:

- people receiving a pharmaceutical intervention;
- people undergoing a surgical intervention;
- pregnant women;
- elite athletes;
- people with any disease who are overweight or obese as a side‐effect of the disease or of a clinical treatment they receive for the disease, such as those with thyroiditis and depression; and
- people with any chronic illness(es);

at baseline and at the post‐intervention phase due to higher or lower health risks compared to the general population. The rationale is that tax policies may affect these subgroups differently from the general population since different causal mechanisms may be operating.

#### Types of interventions

This review included studies of the taxation of unprocessed sugar or sugar‐added foods, defined as:

- a tax of goods;
- enacted by and/or paid to local, regional, or national governments or international organisations;
- of any value or level of taxation;
- added to sales prices of foods with unprocessed sugar or sugar‐added foods, or both (as defined above), and
- implemented for any duration.

Interventions were defined as public policies (i.e. in the form of a tax) of local, regional, national, and multinational governments or field experiments that imitate taxation effects for research purposes in clearly defined environments (e.g. cafeterias, supermarkets and vending machines). A tax was eligible for inclusion if it operated or was payable, or both, at the local, regional, national or international level. We included any comparator intervention (e.g. no intervention, educational interventions, bans, media campaigns, and subsidies on healthy food). We also included studies that compared an eligible tax with another eligible tax that is of a lower value. We have pursued the same strategy in previous Cochrane Reviews on other financial interventions (Pega 2013; Pega 2015; Pega 2017b). We excluded virtual and hypothetical interventions imitating a taxation on unprocessed sugar or sugar‐added foods if participants' purchase decisions are not binding so that they do not all result in a real purchase or if the money is virtual or not belonging to the study participant.

#### Types of outcome measures

Our outcome selection and grouping was guided by preliminary evidence already discussed in the Background and on the basis of the logic model (Figure 2), and incorporated feedback and recommendations from the review advisory board members (email and online survey; Table 4). All pre‐selected outcomes achieved 'critical' or 'important' ratings on average, following the GRADE approach. For primary outcomes we favoured outcomes of critical importance in line with our review scope and Objectives (Table 5). Detailed information on advisory group involvement is provided in the section Searching other resources under the subheading ‘Advisory group’. Primary outcomes include intermediate outcomes directly affected by tax‐induced changes in prices for unprocessed sugar or sugar‐added foods. As a result, consumption of unprocessed sugar or sugar‐added foods may directly alter the primary health outcomes included in this review, including overweight and obesity. Secondary outcomes focused on food patterns (substitution and diet), expenditure, and other prioritised health outcomes directly or indirectly influenced by the taxation of unprocessed sugar or sugar‐added foods. We included demand as a proxy for the consumption of unprocessed sugar or sugar‐added foods.

**2:** Advisory group members

**Name**	**Occupation**
Cristina Cleghorn	Department of Public Health, University of Otago, Wellington, New Zealand
Emilia Crighton	Faculty of Public Health, London, UK
Peter Faassen de Heer	CMO and Public Health Directorate, Scottish Government, Edinburgh, UK
Dionne Mackison	Department for International Development, UK Government, Glasgow, UK
Barry Popkin	Professor of Global Nutrition, University of North Carolina, Chapel Hill, USA
Torben Jørgensen	Professor, Department of Public Health, University of Copenhagen, Copenhagen, Denmark

**3:** Feedback from advisory group (online survey)

**1.1. Rank outcomes according to their relative importance for the scope of the reviews and general public health decision‐making in the context of food taxation^a,b^**		

**Outcomes**	**Average score**	**Rank**
Prevalence of overweight	7.67	3
Prevalence of obesity	7.67	3
Incidence of overweight	8.00	1
Incidence of obesity	8.00	1
Caloric intake through SSBs or unprocessed sugar/sugar‐added foods	7.33	8
Total calorie consumption	6.67	11
Consumption of SSBs or unprocessed sugar/sugar‐added foods (e.g. frequency, amount)	7.33	8
Health‐related quality of life	4.00	16
Total sales of SSBs or unprocessed sugar/sugar‐added foods	5.33	15
Composition of diet (e.g. fat, sugar, salt)	6.67	11
Total expenditure on food	4.00	16
Total expenditure on SSBs or unprocessed sugar/sugar‐added foods (e.g. frequency, amount)	5.67	14
Any health outcomes or health‐related unintended consequences	7.67	3
E.g. mortality	7.00	10
E.g. dental caries	6.00	13
E.g. diabetes	7.67	3
E.g. CVD	7.67	3
**2.1. How well do the presented outcomes cover the basic review scope?**		
**Answers**	**Rating**	**Number of responses**
Important outcomes are presented	66.67%	2
Important outcomes are missing	33.33%	1
Comments (1):	I imagine some evidence will be presented as simply a change in BMI or other markers of obesity rather than a change in incidence or prevalence of obesity (Cristina Cleghorn)	
**3.1. Do you think the same outcomes are appropriate for both reviews (SSB; sugar or sugar‐added foods)?**		
**Answers**	**Rating**	**Number of responses**
The same group of outcomes should be utilised in both reviews	66.67%	2
Different outcomes should be utilised in the two reviews	33.33%	1
Comments (1):	Foods study: hard to go beyond kcal and weight and minimal cardio metabolic outcomes as the Morenga et al. review shows (Barry Popkin)	
**BMI:** body mass index; **CVD:** cardiovascular disease; **SSB:** sugar‐sweetened beverages		

^a^9‐point Likert scale (categories: 1 to 3 – of limited importance; 4 to 6 – important; 7 to 9 – critical). ^b^Three members of the advisory group responded to the survey.

##### Primary outcomes

We included changes from baseline (pre‐intervention) to post‐intervention of the following primary outcomes.

###### Consumption of unprocessed sugar or sugar‐added foods

- consumption of unprocessed sugar or sugar‐added foods (e.g. frequency, amount)

###### Energy intake

- energy intake from unprocessed sugar or sugar‐added foods only
- total energy intake

###### Overweight and obesity

- incidence of overweight
- incidence of obesity
- prevalence of overweight
- prevalence of obesity

Of these outcomes eligible for inclusion in this review, we found evidence on, and were therefore able to include, the outcome of consumption of sugar‐added foods (purchased quantities) as a primary outcome.

##### Secondary outcomes

We considered changes from baseline to post‐intervention of the following secondary outcomes.

###### Substitution and diet

- composition of diet (expressed as food groups or ingredients, e.g. any consumption of any items in the food groups of fats, sugars, salts, and alternative low‐caloric sweeteners), including the consumption of untaxed sugar and sugar‐added foods
- difference in mean consumption of taxed sugar‐added foods compared with untaxed sugar‐added foods

###### Expenditure

- total expenditure on food
- total expenditure on unprocessed sugar or sugar‐added foods
- expenditure on untaxed sugar‐added foods
- difference in mean expenditure on taxed sugar‐added foods compared with untaxed sugar‐added foods

###### Demand

- total sales of unprocessed sugar or sugar‐added foods

###### Other health outcomes

- health‐related quality of life (e.g. Short Form 36 (SF‐36) and Health‐Related Quality of Life (HRQOL‐14))
- mortality
- any other health outcomes (e.g. dental caries, type 2 diabetes, cardiovascular diseases, etc.)

Of the considered secondary outcomes, we only found evidence on, and were therefore able to include in this review, the outcome of expenditure on sugar‐added foods as a secondary outcome.

### Search methods for identification of studies

#### Electronic searches

We searched the following 12 databases:

- Cochrane Central Register of Controlled Trials (CENTRAL; 2019, Issue 10) via Wiley (searched 9 October 2019);
- *Cochrane Database of Systematic Reviews* (CDSR) via Wiley (1995 to 9 October 2019);
- MEDLINE via OvidSP (1946 to 12 September 2019);
- Excerpta Medica database (Embase) via OvidSP (1947 to 12 September 2019);
- PsycINFO via OvidSP (1887 to 9 October 2019);
- Current Contents Medicine Database of German and German‐Language Journals (CCMed) via LIVIVO (1917 to 14 October 2019);
- Latin American and Caribbean Health Sciences (LILACS) via BIREME/VHL (1982 to 12 September 2019);
- EconLit via EBSCO (1969 to 9 October 2019);
- Campbell Library via Campbell Collaboration (2004 to 9 October 2019);
- Food Science and Technology Abstracts (FSTA) via OvidSP (1969 to 14 October 2019);
- Cumulative Index to Nursing and Allied Health Literature (CINAHL) via EBSCO (1937 to 12 September 2019);
- Web of Science (SCI‐EXPANDED, SSCI, A&HCI, CPCI‐S, CPCI‐SSH, ESCI, CCR‐EXPANDED, IC) via Clarivate Analytics (1900 to 12 September 2019).

We applied a search strategy with additional keywords for possible comparators (e.g. 'subsidy') and we did not use filters for study types, in order to maximise the sensitivity of the literature search (Lefebvre 2011, chapter 6.4.4). We used the strategy presented in Appendix 1 to search MEDLINE. We modified this strategy as presented in Appendix 2 to search other electronic databases for records written in any language and published since and until the dates mentioned above. We did not search African Index Medicus (AIM) – a valuable resource for low‐ and middle‐income country literature ‐ in our review, as a sensitive pre‐search with intervention keywords (e.g. tax, taxation, etc.) resulted in zero hits.

We performed one initial search and four search updates in electronic databases.

- We performed an initial search in all electronic databases starting at 27 April 2016.
- We performed a first search update starting at 6 December 2016, searching all electronic databases for records from 27 April 2016.
- We performed a second search update starting at 12 January 2018, searching all electronic databases for records from 6 December 2016.
- When we were close to finalising the review, we performed a last search update, starting at 12 September 2019, for all electronic databases for the most recent publications from 12 January 2018, such as electronic publications ahead of print.

##### Grey literature databases

We searched the following six grey literature databases with search strategies as presented in Appendix 3.

- ProQuest Dissertations & Theses Database (PQDT): UK and Ireland via ProQuest (1637 to 9 October 2019);
- System for Information on Grey Literature in Europe – OpenGrey via OpenGrey (1994 to 9 October 2019);
- The Directory of Open Access Repositories – OpenDOAR via OpenDOAR (1739 to 12 December 2016, database not accessible in subsequent searches);
- EconPapers via REPEC (1997 to 14 October 2019);
- Social Science Research Network – SSRN eLibrary via SSRN (1994 to 14 October 2019);
- National Bureau of Economic Research (NBER) via NBER (1920 to 13 October 2019).

We performed an initial search in all grey literature databases starting at 27 April 2016 and applied the same search time frames for updates as described for the electronic databases.

We searched the following two databases for completed or ongoing studies with keywords relevant to the intervention (e.g. taxation, pricing):

- WHO International Clinical Trials Registry Platform (WHO ICTRP; includes references of the ClinicalTrials.gov database) via WHO (1988 to 14 October 2019); and
- Trials Register of Promoting Health Interventions (TRoPHI) via EPPI‐Centre (2004 to 11 August 2016, free text search not accessible in subsequent searches).

##### Internet search engines

We screened the first 30 hits in Google Scholar via Google on 11 August 2016 and 14 October 2019. The search strategy is presented in Appendix 4.

##### Targeted internet searching of key organisational and institutional websites

We searched websites of major organisations and institutions in the initial search in 2016 and on 11 October 2019, specifically:

- World Obesity Federation (www.worldobesity.org);
- OECD (www.oecd.org);
- European Commission (ec.europa.eu/index_en.htm);
- DG Sanco (ec.europa.eu/dgs/health_food‐safety/index_en.htm);
- Centers for Disease Control and Prevention (www.cdc.gov);
- National Institute for Health and Care Excellence (www.nice.org.uk);
- World Trade Organization (www.wto.org);
- World Cancer Research Fund Institute (www.wto.org); and
- WHO (www.who.int).

#### Searching other resources

We handsearched the reference lists of all included studies.

##### Advisory group

We established a review advisory group of experts in the field of food taxation and health to comment and provide advice and suggestions to improve the systematic review and its manuscript at the protocol stage. Following the GRADE approach, the advisory group members participated in an online survey and ranked pre‐selected outcomes according to their relative importance on a 9‐point Likert scale (categories 1 to 3: of limited importance; 4 to 6: important; 7 to 9: critical; GRADE 2013). The review advisory group consisted of policy makers, researchers and academics.

We provided the members of the review advisory group with detailed background information on this review. At the protocol stage, the review advisory group members were asked to provide feedback specifically on the focus and relevance of this review’s research question, selected outcomes, study design, search strategy, database selection, and ongoing or unpublished studies (Green 2011, chapter 2.3.4.3). In the review stage, prior to final submission, we contacted review advisory board members for relevant ongoing and unpublished studies. We received feedback via email and the online survey. All members of the advisory group and results from the online survey are found in Table 4 and Table 5.

### Data collection and analysis

#### Selection of studies

An information specialist (CF) and an additional author (TLH) conducted the electronic database searches, searches within grey literature databases and internet search engines. One review author (MP) handsearched the reference lists of included studies. We performed targeted internet searching of key organisational and institutional websites, using a standardised template to document the search (MP, THL, SKL, UG, GG, FP, IS, SVK).

We conducted screening in six stages. If a reference, an abstract or a full‐text report was in a language other than English, German or French, we translated it using internet‐based translation tools or by asking native speakers. First, at least two review authors (MP, TLH, SKL, UG, GG, FP, IS or SVK prior to 2018; MP, TLH or SKL in 2018 and 2019) independently screened studies’ titles and abstracts (when available). MP, THL and SKL provided a detailed screening guideline for all review authors and used Covidence for screening titles and abstracts (MP, TLH, SKL, UG, GG, FP, IS, SVK). If an abstract was not provided by the database it originated from, and the title appeared to be potentially relevant, we progressed the record to full‐text review within Covidence. Second, we resolved disagreement by consensus and in consultation with a third review author (SKL, TLH or MP) and eliminated all records that did not fit the inclusion criteria (see Criteria for considering studies for this review). Third, we retrieved full texts of potentially relevant studies for assessment. Fourth, two review authors (MP and TLH) independently screened the full texts. Fifth, both review authors created a list of studies that they considered to fulfil the inclusion criteria. Sixth, the review authors compared their lists and in cases of disagreement, a third review author (SKL) was decisive. Based on these six steps, we included studies in the review. At each stage, we recorded the records retrieved and excluded. For key records of which we screened the full texts, we recorded reasons for exclusion. We present a PRISMA flowchart in Figure 3 to display the selection of included studies (Liberati 2009).

**3:**
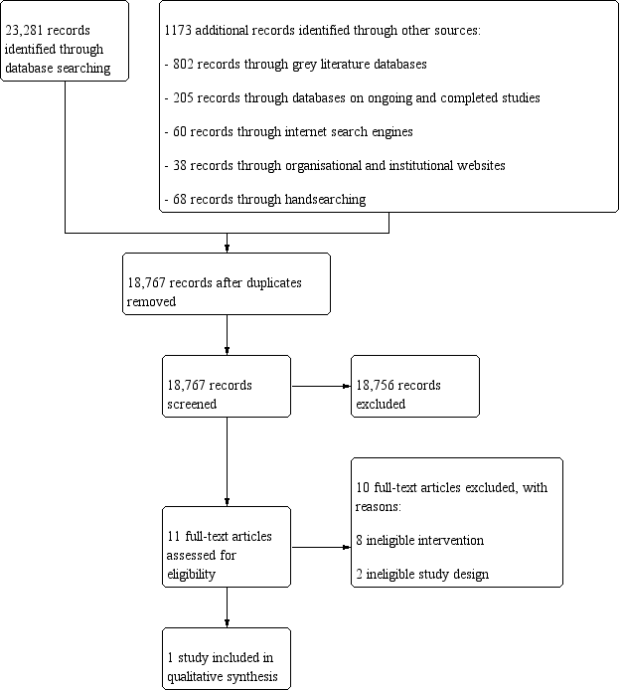
Study flow diagram

#### Data extraction and management

We stored all records obtained by the electronic searches in a reference management software (Endnote 2012). We recorded and managed the results of the abstract and full‐text screening. MP, SVK, MHB, TLH and SKL further discussed the reasons for exclusion at full‐text assessment and we stored results in an Excel spreadsheet. We used a modified data extraction and assessment template from Cochrane Public Health (CPH; CPH 2011), for the complex intervention addressed in this review. We extracted effect estimates for study populations based on PROGRESS categories (place of residence, race/ethnicity/culture/language, occupation, gender/sex, religion, education, socioeconomic status, social capital) to evaluate impacts on equity. We considered the Cochrane & Campbell Methods Equity Checklist (CCEMG 2012).

At least two review authors (MP, SVK, and FP prior to 2018; MP and MHB in 2018 and 2019) independently extracted data and both compared the extracted data. A third review author (SKL or TH) resolved disagreements. Prior to the main data extraction process, MP, TLH, SVK, UG, FP, and SKL piloted and adapted the data extraction form to ensure standardised extraction (Higgins 2011a, chapter 7.6.3). MP, SVK, and FP (prior to 2018), and MHB (in 2019) extracted general information (publication type, country of study, funding source of study, potential conflict of interest), study eligibility (type of study, participants, type of intervention, duration of intervention, and type of outcome measures), study details (study aim, methods, results, intervention group, confounders, and confounder‐adjusted and unadjusted outcomes), indicators of changes in food prices (price of unprocessed sugar or sugar‐added foods, price of other food categories), and other relevant information. We also extracted contextual factors (e.g. political system, co‐interventions, reason for implementation, reason for particular tax level, intended beneficiaries, implementation costs, country and region‐specific level of gross domestic product (GDP), food security (availability, access, and use), and process evaluation criteria (e.g. satisfaction of participants, adherence) that facilitate or hinder the implementation of taxation on unprocessed sugar or sugar‐added foods (Anderson 2011a; Campbell 2018). If studies did not provide information on these criteria but referred to another study, we extracted information from these other sources. In the Characteristics of included studies we described methods, participants, interventions, outcomes and further notes. We did not extract qualitative data.

MP entered, stored and managed extracted data in Review Manager 5 and MHB double‐checked the data entered (Review Manager 2014).

#### Assessment of risk of bias in included studies

Two review authors (MP, FP and SVK prior to 2018; MP and MHB in 2018 and 2019) independently evaluated the risk of bias of every included study. In case of disagreement, they discussed discrepancies with a third review author (TLH or SKL) and resolved them by consensus. Based on the template provided by CPH, we assessed the risk of bias using the criteria for judging risk of bias in Cochrane's 'Risk of bias' assessment tool (Higgins 2011), and Cochrane EPOC's guidance (EPOC 2015). Both tools examine the following biases: selection, performance, detection, attrition, reporting, and others. The EPOC 'Risk of bias' tool for ITS examines three further risks of bias: was the intervention independent of other changes, was the shape of the intervention effect pre‐specified and was the intervention unlikely to affect data collection? For studies included in the main quantitative evidence synthesis (i.e. RCTs, cRCTs, nRCTs, CBA and ITS studies), we planned to assess the risk of bias using the 'Risk of bias' criteria for EPOC reviews, based on the Cochrane tool for assessing risk of bias (Higgins 2011b, Table 8.5.a).

We planned to assess the study quality and risk of bias of 'supporting studies' (i.e. studies that do not meet EPOC criteria, cohort studies, repeated cross‐sectional studies, UBA studies) with the Quality Assessment Tool for Quantitative Studies, developed by the Effective Public Health Practice Project (EPHPP) (EPHPP 2007).

To judge the risk of bias according to Cochrane's 'Risk of bias' assessment tool, we used the following three ratings: 'low', 'high', and 'unclear' (adequate information is unavailable or there is uncertainty about the risk of bias; Higgins 2011b, chapter 8.6). For studies not meeting EPOC criteria, we planned to judge the risk of bias according to the Quality Assessment Tool for Quantitative Studies, using following three categories: 'strong', 'moderate', and 'weak' (EPHPP 2007). We provide 'Risk of bias' tables for all included studies in the Results section.

#### Measures of treatment effect

In the data synthesis, we aimed to quantitatively pool the results of different studies using meta‐analysis. However, since we were not able to perform meta‐analyses, we have not presented a pooled effect estimate in the systematic review.

The included studies reported treatment effects of a tax on sugar‐added foods for consumption (purchased quantities) and expenditure, with the outcomes measured using continuous data, and the treatment effect measures being a standardised mean difference (SMD) with standard error (SE). We calculated the standard deviations (SDs): SD = SE* √n where SE= standard error, and n = number of household‐level observations. Then, we calculated the 95% confidence interval (CI) of the effect estimate: μ = *M* ± *T*(*s_M_*) where *M* = sample mean, *T* = T statistic determined by confidence level (here: 95%) and *s_M_* = standard error = √(*s*^2^/*n*).

We did not find evidence on the effects of the treatment (i.e. tax intervention) on any dichotomous outcomes, and thus we have not reported any relative or absolute measures of treatment effect for dichotomous outcomes (e.g. odds ratios (ORs), risk ratios (RRs) or risk differences (RDs)).

#### Unit of analysis issues

As per Cochrane guidelines, we planned to collect data on allocation and to analyse the level at which allocation occurred for the same outcome (Deeks 2011, chapter 9.3.1). We considered data from cross‐over trials (e.g. by incorporating the study data similar to a parallel‐group trial) and studies with multiple observations (e.g. by defining different periods of follow‐up) for potential analyses (Deeks 2011, chapter 9.3.4; Higgins 2011c, chapter 16.4.5).

We planned to request individual‐level data from the corresponding author of the study if control for clustering was missing or insufficient and if individual‐level data were not presented in the study. We planned to reduce the size of each trial to its 'effective sample size' in order to correct intervention effects in cluster‐randomised trials. The effective sample size of an intervention group is the original sample size divided by the 'design effect'. We planned to calculate the design effect with the formula 1 + (M – 1) ICC, where M is the average cluster size and ICC is the intracluster correlation coefficient (Higgins 2011c, chapter 16.3.4).

For dichotomous data, we planned to divide the total number of participants and the number of participants who experienced the event by the same design effect. For continuous data, we planned to reduce only the sample size, with means and standard deviations to remain unchanged (Higgins 2011c, chapter 16.3.4).

We included only one study, and thus, it was not feasible to perform analyses on the level at which allocation occurred (e.g. for multiple interventions).

#### Dealing with missing data

We planned to request all missing information and data from principal study authors via email. In the study from Biró 2015, data are based on a household‐level survey not conducted by the principal study author. The number of distinct households and participants was not available in the study from Biró 2015. Therefore, and for further methodological issues, we contacted the study author via email. We received responses via email, but the number of distinct households and participants could not be clarified and thus, in agreement with the study author, we refer to household‐level observations.

#### Assessment of heterogeneity

We were not able to perform meta‐analyses for the outcomes because only one study was included in this review. There were not enough studies included to assess heterogeneity across studies regarding potential sources of heterogeneity, such as study population, intervention area/setting, intervention characteristics (tax definition, basis for calculating taxation, level of taxation), implementation level, comparisons, co‐interventions, and outcomes.

#### Assessment of reporting biases

Reporting bias, including publication bias, time lag bias, multiple (duplicate) publication bias, location bias, citation bias, language bias, and outcome reporting bias occurs when the dissemination of research results depends on their magnitude or direction, or both (Sterne 2011). To assess the presence of reporting bias, we planned to produce funnel plots if we found 10 or more studies of the same outcome. Had we found 10 or more studies to include, then we would have tested for asymmetry in funnel plots (small study effects) by investigating whether the relationship between a measure of study size and the estimated intervention effect is asymmetrical (Sterne 2011). However, since the review included fewer than 10 studies of the same outcome, we did not assess reporting bias.

#### Data synthesis

As already described, we could not perform meta‐analyses with one included study. We narratively summarised the study results. We structured the summary by the outcome categories of this review. Within these categories, we planned to make further separation according to the intervention setting and the study design or study quality (Ryan 2016). However, this was not feasible due to the inclusion of only a single study. In addition to reporting findings as text and tables, we considered both harvest plots and effect direction plots to summarise data not suitable for meta‐analyses. Harvest plots are graphical summaries of data represented by multiple shaded or non‐shaded bars with varying heights, and can be utilised to indicate effect directions across included studies with non‐standardised effect estimates of outcomes (e.g. anthropometric measures). Similarly, effect direction plots can be used to visualise information on effect directions, with more focus on direct comparisons across studies (Ogilvie 2008; Thomson 2013). However, as we have only included one study in this review, we did not represent data by harvest plots or effect direction plots.

For reports of multiple follow‐ups for the same outcome (e.g. six months during the intervention, one year during the intervention, and six months after the end of the intervention), we planned to prioritise the longest follow‐up during the intervention (e.g. one year during the intervention, in the example given). However, in the included study, data of only one follow‐up were available.

We planned to map results of the data synthesis against our initial logic model, to refine the theory of change and assess the credibility of the assumed causal pathways (Anderson 2011a; Thomson 2013). Due to limited results, we have illustrated the mapped and unmapped causes, effects and outcomes within the included study in an adapted logic model with causal pathways in Figure 4.

**4:**
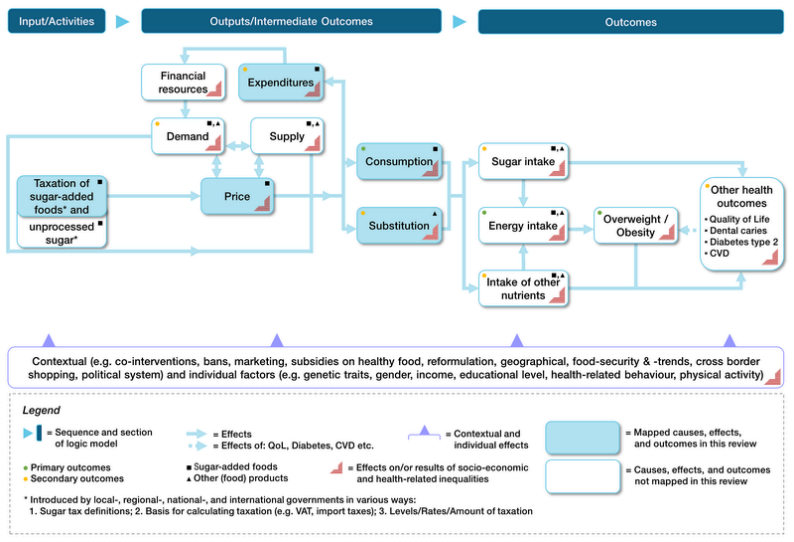
Study's adapted logic model with causal pathways

#### Subgroup analysis and investigation of heterogeneity

Due to the inclusion of only one study in this review, it was not feasible to conduct meta‐analyses or produce harvest plots for primary outcomes with subgroups of interest.

We planned to run subgroup analyses for primary outcomes with regard to:

- high‐income countries versus middle‐ and low‐income countries;
- high‐income groups versus middle‐ and low‐income groups;
- high‐educated groups versus low‐educated groups;
- different tax values of unprocessed sugar or sugar‐added foods;
- single tax on unprocessed sugar or sugar‐added foods versus multiple taxes on unprocessed sugar or sugar‐added foods;
- tax on unprocessed sugar or sugar‐added foods alone versus tax on unprocessed sugar or sugar‐added foods accompanied by other fat taxes or interventions (e.g. bans, minimum pricing, media campaigns, or subsidies on healthy foods);
- different types of taxation:
  - indirect taxes levied within national borders (e.g. excise tax, sales tax, value added tax (VAT)); and
  - import (or export) taxes including custom duties and import sales taxes;
- children versus adults;
- BMI subgroups;
- indigenous populations;
- chronically ill people with overweight and obesity as side‐effects.

We planned to perform subgroup analyses with data on PROGRESS categories (e.g. age, gender, education, and ethnicity; Anderson 2011b).

Finally, we planned to investigate the statistical significance of differences in the treatment effect between subgroups using t‐tests and Chi² tests (Deeks 2011, chapter 9.6.2).

#### Sensitivity analysis

We planned to perform sensitivity analyses to determine the robustness of our results by conducting meta‐analyses and harvest plots for the studies included in our review:

- with respect to source of funding;
- with studies considered as ‘low risk of bias’ compared to studies considered as ‘high risk of bias’;
- with published versus unpublished studies;
- with respect to the intervention duration;
- with respect to follow‐up time;
- with objective measures versus subjective measures;
- with respect to study design;
- with respect to cut‐off points of the measures of treatment effect;
- with respect to imputation of data.

We planned not to include studies in sensitivity analyses if the studies had a high or unclear risk of bias with respect to incomplete outcome data or baseline differences. For cRCTs with adequate data provided, we planned to perform intracluster correlation value sensitivity analysis. We planned to report findings of sensitivity analyses as a summary table (Deeks 2011, chapter 9.7).

However, since we did not perform a meta‐analysis, none of the planned sensitivity analyses were feasible.

##### Summary of findings and assessment of the certainty of the evidence

###### Summary of findings

We have provided two 'Summary of findings' tables, one for primary outcomes and one for secondary outcomes (Schünemann 2011, chapter 11.5). As suggested by an external referee, we reported the following pre‐selected outcomes from included studies: consumption of unprocessed sugar and sugar‐added foods (purchased quantities; primary outcome) and expenditure on unprocessed sugar and sugar‐added foods (secondary outcome).

###### GRADE

For each outcome, two review authors (MP, MHB) assessed the certainty of the evidence for the domains 'risk of bias', 'inconsistency', 'indirectness', 'imprecision', and 'publication bias'. Table 1 includes information on the primary outcomes; the summary of findings on reported secondary outcomes is included in Table 2. Both the tables include anticipated absolute effects, the number of participants, the number of studies included, the certainty of evidence based on the GRADE guidelines, and additional comments. We used the computer software GRADEpro GDT to prepare the 'Summary of findings' table. As we included only one study, we have provided GRADE considerations narratively, in accordance with Cochrane MECIR standards for the inclusion of one single study (Higgins 2019).

## Results

### Description of studies

#### Results of the search

Figure 3 is a PRISMA flowchart, demonstrating the search results from databases and other sources of literature. In October 2019, we completed the literature search for potentially relevant studies in 12 electronic databases, six grey literature databases, two databases for completed or ongoing studies, one internet search engine, and 10 key organisational and institutional websites, as well as handsearching of reference lists. We retrieved a total of 24,454 records.

In total, we performed searches at four intervals, including one initial search starting in April 2016, yielding 17,080 records, and search updates starting in December 2016 (1492 records), January 2018 (2253 records), and September 2019 (3629 records).

Altogether, we retrieved 23,281 records from the 12 electronic databases, 802 records through grey literature databases, 205 records through databases for completed or ongoing studies, 60 records through internet search engine searches, 38 records through organisational and institutional websites and 68 records through handsearching. After removing duplicates of the records retrieved from different sources, a total of 18,767 records remained, for which we screened titles and abstracts, using the computer program Covidence. In the process of title and abstract screening, we excluded 18,756 records, resulting in 11 records that we considered potentially eligible for inclusion. Of these 11 records, we screened full texts, excluding 10 studies (see Excluded studies), resulting in one study (Biró 2015), fulfilling the inclusion criteria of this review. Thus, we included the Biró 2015 study in our review.

#### Included studies

According to our eligibility criteria, we included one study with a total of 44,608 household‐level observations over five survey waves (Biró 2015). The study reported 40,210 household‐level observations for the primary and secondary outcomes of interest. Information on the study's methods, participants, interventions, outcomes and sources of funding are given in the Characteristics of included studies tables.

##### Funding

Biró 2015 was funded by the Scottish Institute for Research in Economics (SIRE) Early Career Engagement Grant. The Hungarian Central Statistical Office provided access to the data. The study author states that the views expressed in the study do not in any way represent the views of the Hungarian Central Statistical Office.

##### Study types and methods

###### Study type

####### Interrupted time series (ITS)

The included study is an ITS study, meeting EPOC criteria for study inclusion in a review (EPOC 2012). ITS studies are non‐randomised study designs. In the included study, large‐scale panel data are taken from the Hungarian Household Budget and Living Conditions Survey from the years 2008 to 2012 (five waves). Data were collected on a monthly basis. Beginning 1 September 2011, Hungarians had to pay a content‐based tax on specific food categories high in sugar, salt, and caffeine. Details on the taxed products and the taxation level are provided in Table 6 as outlined by Biró 2015 and Martos 2017. Thus, from January 2008 to August 2011, there are 44 time points of pre‐intervention measurements. Data from September 2011 to December 2012 include 16 months, i.e. 16 time points, of post‐intervention measurements. As the so‐called 'public health product tax' was legislated by the Hungarian Parliament and implemented at the national level, all participants of Biró 2015 received the intervention.

**4:** Hungarian public health product tax

**Taxed products**	**Threshold levels**	**Tax rate (HUF per litre or kg)**	
		**1 September 2011**	**1 January 2012**
SSBs	> 8 g sugar/100 mL	5	7
SSB concentrates and syrups	> 8 g sugar/100 mL and fruit < 25%	‐	200
Energy drinks	> 1 mg methylxanthines/100 mL or > 100 mg taurine/100 mL	‐	250
Energy drinks	> 8 g sugar/100 mL or caffeine > 10 mg/100 mL	250	‐
**Pre‐packaged products with added sugar**	**Total sugar > 25 g/100 g**	**100**	**130**
**Chocolates**	**> 40 g sugar/100 g and < 40 g cocoa/100 g**	**100**	**130**
**Sugar‐sweetened cocoa powder**	**> 40 g sugar/100 g and < 40 g cocoa/100 g**	‐	**70**
Salted snacks	> 1 g salt/100 g	200	250
Condiments (some exemptions for mustards, ketchups)	> 5 g salt/100 g	200	250
Flavoured beer or alcoholic drink	> 5 g sugar/100 mL	‐	20
**Fruit preserves, jam, excluding 'extra' versions**	**> 35 g sugar/100 g**	‐	**500**
**HUF:** Hungarian Forint; **SSB:** sugar‐sweetened beverage;			

This is table is derived from Biró 2015 and Martos 2016.

###### Study methods

Analytical methods applied in Biró 2015 include regression analysis with fixed‐effect models (see Gunasekara 2014 for description of these models), using large‐scale panel data from the Hungarian Household Budget and Living Conditions Survey from the years 2008 to 2012. Treatment effects were estimated with SMDs with SEs. The regression analyses included household fixed‐effect and linear trends in all models. Household characteristics, as described by Biró 2015, included living area, whether the head of the household was at least a high school graduate, age composition and average age of the household, activity and average subjective health of the household, number of household members, income decile the household belonged to, and three indicators of financial well‐being. The study assessed the effectiveness of the Hungarian public health product tax on the consumption and expenditure of sugar‐added foods (taxed sweets, untaxed sweets and differences between taxed and untaxed sweets) and other food categories high in salt and caffeine. We considered the effect of the tax on sugar‐added foods (as part of the Hungarian public health product tax) on consumption and expenditure of these foods specifically. The quantity of consumed taxed and untaxed sugar‐added foods is measured in kg. The quantity of expenditure is nominal. Results on the intervention effects are based on 42,100 observations. We did not find any study that provided evidence on the health, consumption, substitution, and expenditure effects of the taxation of unprocessed sugar.

##### Participants

Biró 2015 describes the study population as follows: "The annual sample covers around 10 thousand households, 26 thousand individuals. This gives overall 44,608 household level observations throughout the 5 survey waves. The survey is a 4‐years rotational survey: each household remains in the survey for 4 years" (p.110). The survey was set up in 2005. Thus, data from 2008 belong to the first four years of data collection and therefore, data from 2008 contain information from different households and individuals than data from 2009 to 2012.

##### Interventions

###### Taxation of unprocessed sugar

We did not identify any studies on the taxation of unprocessed sugar.

###### Taxation of sugar‐added foods

Biró 2015 addressed the Hungarian public health product tax that came into effect in September 2011 and was modified in January 2012. We have provided detailed information on the taxed products, the sugar threshold levels and the tax rate of the Hungarian public health product tax in Table 6. The Hungarian public health product tax includes the taxation of foods with a specific sugar content. However, the intervention also includes the taxation of SSBs and foods high in sugar, and caffeine. As the government implemented this intervention at the national level, the intervention was universal, covering the whole population.

##### Outcomes

###### Primary outcome

The included Biró 2015 study reported on one primary outcome: the consumption of sugar‐added foods (purchased quantities). The intervention was not applied to the total of all sugar‐added foods, but to the following specific categories only:

- pre‐packaged products with added sugar (total sugar content more than 25 g per 100 g)
- chocolates (sugar content more than 40 g per 100 g and cocoa content less than 40 g per 100 g)
- sugar‐sweetened cocoa powder (sugar content more than 40 g per 100 g and cocoa content less than 40 g per 100 g)
- jam (sugar content more than 35 g per 100 g).

Untaxed sugar‐added foods primarily include fresh confectionary, fresh bakery products, and sugar‐added foods from the categories above with lower sugar levels.

Biró 2015 analysed all of these taxed sugar‐added foods under the category of 'taxed sweets', whereas it categorised the sugar‐added foods that were not captured by the tax as 'untaxed sweets'. In our review, we have therefore included the mean consumption of taxed sugar‐added foods as primary outcome.

Biró 2015 measured this outcome at the household level. They derived measures from a monthly consumption diary. These were self‐reported measures. They assessed the outcome continuously on the basis of consumption diaries, starting 44 months before the implementation of the tax until 16 months after the implementation of the intervention.

###### Secondary outcome

Biró 2015 reported five secondary outcomes related to substitution and expenditure on sugar‐added foods.

- Substitution. Consumption of untaxed sugar‐added foods and the difference in the consumption of taxed sugar‐added foods as compared to untaxed sugar‐added foods are considered as a direct measurement of substitution and an indicator of the strength of substitution, respectively:
  - mean consumption of untaxed sugar‐added foods; and
  - difference in the mean consumption of taxed sugar‐added foods, compared with untaxed sugar‐added foods.
- Expenditure:
  - mean expenditure on taxed sugar‐added foods;
  - mean expenditure on untaxed sugar‐added foods; and
  - difference in the mean expenditure on taxed sugar‐added foods, compared with untaxed sugar‐added foods.

Biró 2015 measured these outcomes at the household level; measures were from a monthly consumption diary and they were self‐reported. They assessed these outcomes continuously on the basis of consumption diaries, starting 44 months before the implementation of the tax until 16 months after the implementation of the intervention.

#### Excluded studies

We screened full texts from a total of 11 potentially relevant studies. Ten of these studies did not fulfil our a priori‐defined eligibility criteria for this review. In the Characteristics of excluded studies table, we describe the reasons for their exclusion from this review. In all excluded studies the intervention was ineligible. Such ineligible interventions were, for example, the taxation of food categories according to a pre‐defined caloric content that consists of different high caloric ingredients, such as fat, sugar, and carbohydrates (Batis 2016; Mauricio 2019; Taillie 2017). Taxation of energy‐dense and high‐caloric foods does not contain a minimum threshold value on the content of sugar per 100 g or per kg: this type of intervention and its effect is therefore not attributable to the taxation of unprocessed sugar or sugar‐added foods.

We excluded the study design 'simulation study' and studies with ineligible study outcomes from our review. Bridgman 2007, for example, was consequently excluded from this review for both reasons.

##### Ongoing studies

We did not identify any ongoing studies.

### Risk of bias in included studies

For the included ITS design, we have presented details for the risk of bias in Figure 5. Further details are provided in the tables of the section Characteristics of included studies.

**5:**
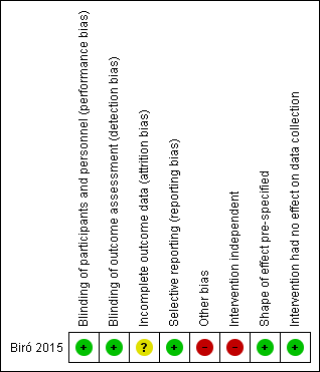
Risk of bias summary for ITS study

In summary, we judged Biró 2015 at low risk of bias in five domains, unclear risk of bias in one domain and high risk of bias in two domains. Given the limited number of outcomes, we conducted the 'Risk of bias' assessment at the level of the study as a whole.

#### Allocation

In ITS designs, generation of allocation sequence and adequate concealment of allocation sequence is not applicable.

#### Blinding

##### Blinding participants and personnel (performance bias)

Biró 2015 used data from the Hungarian Household Budget and Living Conditions Survey. All participants in this household survey received the intervention, since the Hungarian public health product tax was implemented at the national level. The participants and the personnel in the Hungarian Household Budget and Living Conditions Survey, however, did not know that consumer data would subsequently be used to estimate the health and expenditure effects of the Hungarian public health product tax. Although neither participants nor personnel were blinded, we rated the risk for performance bias as low, because the participants could not know that their information would later be used to measure the effect of the Hungarian public health product tax, especially since data collection started six years prior to the implementation of the Hungarian public health product tax. Thus, we believe that knowledge about the intervention did not affect participants' and personnel's reporting behaviour and therefore, it may not have influenced the outcome.

##### Blinding outcome assessment (detection bias)

The outcomes assessed, expenditure and purchased quantities of sugar‐added foods, were self‐reported by the survey participants in the same month of different survey waves. Expenditure and purchased quantities of sugar‐added foods were self‐reported subjective measures. Self‐reporting of outcomes is likely to bias the results in unknown ways. However, all participants received the intervention and the participants did not know that their data would later be used to assess the effect of the Hungarian public health product tax on expenditure and purchased quantities of sugar‐added foods. The survey was not linked to sugar consumption at all. Therefore, we rated the risk of detection bias for blinding of outcome assessors as low, as participants' knowledge on the intervention was unlikely to have biased the results.

#### Incomplete outcome data

Biró 2015 did not report information on incomplete outcome data. As data were used from the Hungarian Household Budget and Living Conditions Survey administered by the Hungarian Central Statistical Office, we searched the statistical office's website and publications for information on the rates of study non‐participation, item non‐response, loss to follow‐up and methods used for handling missing data. We did not find any such information and therefore rated attrition bias as unclear.

#### Selective reporting

ITS (observational) studies of household‐level data do not generally provide study protocols or trial registrations. The included ITS study (Biró 2015) did report all expected outcomes and presented complete data and additional analyses in a supplement. We did not find evidence of selective reporting and therefore rated the risk of reporting bias as low.

#### Other potential sources of bias

##### Freedom from other risks of bias

Biró 2015 derived data from the Hungarian Household Budget and Living Conditions Survey. As Biró 2015 reported, while this survey did collect data on expenditure on different foods and on quantities of different foods purchased, it did not collect data on the exact quantity of sugar in these different foods, for example sugar content in a jam purchased. Therefore, it is very likely that untaxed foods were misclassified as taxed foods, and vice versa. This may have biased the results of the outcomes, leading probably to an underestimate of the effect. Therefore, we judged the risk of other bias (here: misclassification of the outcome) as high.

##### Interventions independent

On 1 September 2011, the Hungarian government implemented the public health product tax, a content‐based tax on specific food categories high in sugar (including SSBs), salt, and caffeine, which was revised on 1 January 2012 (including tax increases). Therefore, the taxation of sugar‐added foods was accompanied by the taxation of SSBs, salt and caffeine. Table 6 provides an overview of the implementation of the Hungarian public health product taxes on 1 September 2011 and 1 January 2012 (Biró 2015; Martos 2017), and how this intervention implementation corresponds with the different waves of data collection used in the included study (Biró 2015). The taxation of sugar‐added foods was not fully independent of other changes, because the other co‐interventions implemented in parallel (i.e. taxation of SSBs and products high in salt and caffeine) may have influenced our outcomes, in unknown ways. We rated the risk of bias from co‐interventions as high.

##### Shape of effect pre‐specified

Although the time of analysis was not the same time as the point of intervention, we judged the risk of bias to be low, as the change in the effects occurred in a plausible timeframe and manner. Biró 2015 included time as a variable in the analysis with a time‐specific indicator of taxation.

##### Intervention had no effect on data collection

Biró 2015 used data from the Hungarian Household Budget and Living Conditions Survey, which was conducted fully independently of the implementation of the Hungarian public health product tax. The same methods of data collection were applied pre‐ and post‐intervention and we consequently rated the risk of bias from the intervention affecting data collection as low.

### Effects of interventions

See: Table 1; Table 2

Table 1 presents an overview of the effects of the taxation of sugar‐added foods for the primary outcome. Table 2 presents an overview of the effects of the taxation of sugar‐added foods for reported secondary outcomes.

#### Primary outcomes

##### Consumption of unprocessed sugar or sugar‐added foods

Biró 2015 did not measure consumption of unprocessed sugar. However, the study provided evidence on the effects of taxing foods exceeding a specific sugar threshold value on the consumption of sugar‐added foods.

###### Consumption of taxed sugar‐added foods

Biró 2015 provided evidence on the effect of taxing foods exceeding a specific sugar threshold value on the consumption of sugar‐added foods. After implementation of the Hungarian public health product tax, the mean consumption of taxed sugar‐added foods (measured in units of kg) decreased by 4.0%, corresponding to a reduction of 40 g per kilo (SMD −0.040, 95% CI −0.07 to −0.01; P < 0.05; SE 0.02; SD 3.41; 40,210 households; very low‐certainty evidence).

##### Energy intake

The included study did not measure energy intake through unprocessed sugar or sugar‐added foods, or total energy intake.

##### Overweight and obesity

The included study did not measure the incidence or prevalence of overweight or obesity.

#### Secondary outcomes

##### Substitution and diet

###### Consumption of untaxed sugar‐added foods

The mean consumption of untaxed sugar‐added foods (measured in units of kg) decreased after implementation of the tax intervention by 1.3%, corresponding to a reduction of 13 g per kg (SMD −0.013, 95% CI −0.05 to 0.02; P > 0.10; SE 0.02; SD 3.41; 40,210 households; very low‐certainty evidence).

###### Difference in the consumption of taxed sugar‐added foods compared with untaxed sugar‐added foods

The mean consumption of taxed sugar‐added foods (measured in units of kg) did not differ meaningfully from the mean consumption of untaxed sugar‐added foods after the implementation of the intervention (SMD −0.028, 95% CI −0.07 to 0.02; P > 0.10; SE 0.02; SD 4.61; 40,210 households; very low‐certainty evidence).

##### Expenditure

Biró 2015 did not measure total expenditure on food or total expenditure on sugar. However, the study provided evidence on the effects of taxing foods exceeding a specific sugar threshold value on the mean expenditure on sugar‐added foods.

###### Expenditure on taxed sugar‐added foods

There was no effect of the intervention on the mean expenditure on taxed sugar‐added foods (measured in units of Hungarian Forint (HUF)), although data show that the mean expenditure decreased by 0.6% (SMD −0.006, 95% CI −0.03 to 0.02; P > 0.10; SE 0.01, SD 2.81; 40.210 households; very low‐certainty evidence).

###### Expenditure on untaxed sugar‐added foods

The mean expenditure on untaxed sugar‐added foods (measured in units of HUF) increased after the implementation of the Hungarian public health product tax by 3.0% (SMD 0.03, 95% CI −0.01 to 0.07; P < 0.10; SE 0.02; SD 3.61; 40.210 households; very low‐certainty evidence).

###### Difference in the expenditure on taxed sugar‐added foods compared with untaxed sugar‐added foods

The mean expenditure on taxed sugar‐added foods (measured in units of HUF) differs from the mean expenditure on untaxed sugar‐added foods by −3.7% (SMD −0.037, 95% CI −0.08 to 0.01; P < 0.10; SE 0.02; SD 4.41; 40.210 households; very low‐certainty evidence).

##### Demand

The included study did not measure the total sales of unprocessed sugar or sugar‐added foods.

##### Other health outcomes

The included study did not measure health‐related quality of life (e.g. Short Form 36 (SF‐36), Health‐Related Quality of Life (HRQOL‐14)), mortality, or any other health outcomes (e.g. dental caries, type 2 diabetes, cardiovascular diseases, etc.).

## Discussion

### Summary of main results

From a total of 24,454 records, one study (Biró 2015), met the a priori‐defined eligibility criteria for inclusion in our systematic review. We identified evidence on the effects of taxing sugar‐added foods regarding their consumption (primary outcome), substitution and expenditure (secondary outcomes). However, we found no studies that looked at the effects of taxing sugar‐added foods on other consumption‐related outcomes such as energy intake, or on other expenditure‐related outcomes, such as total sales of sugar‐added foods. Moreover, we found no studies that looked at the effects of taxing sugar‐added foods on health‐related outcomes, such as overweight, obesity and other health outcomes. Findings from our systematic review show that there is a substantial lack of evidence on the effects of taxing unprocessed sugar as we did not identify any study investigating this kind of intervention and its effects.

From the results of this systematic review, as derived from one included study, we do not know whether the taxation of sugar‐added foods is effective for reducing their consumption. The results from the primary study indicated a small reduction in consumption, but the certainty of the evidence is very low. The effect on the mean consumption on untaxed sugar‐added foods was small and inverse, that is, not reflecting a substitution effect. However, regarding the very low certainty of the evidence, we do not know whether the taxation of sugar‐added foods in fact results in a substitution effect or not. Also, the effect of taxing sugar‐added foods on the difference in the consumption of taxed sugar‐added foods as compared to untaxed sugar‐added foods is considered to be small. However, we are uncertain if taxing sugar‐added foods has an effect on the difference in the consumption of taxed sugar‐added foods as compared to untaxed sugar‐added foods. We do not know whether the taxation of sugar‐added foods is effective for reducing expenditure on taxed sugar‐added foods. Although our single included study showed an effect of taxing sugar‐added foods on the expenditure on untaxed sugar‐added foods and the effect on the difference in the expenditure on taxed sugar‐added foods as compared to untaxed sugar‐added foods, the certainty of the evidence is very low. Therefore, we are uncertain whether taxing sugar‐added foods increases expenditure of untaxed sugar‐added foods and affects the difference in the expenditure of taxed sugar‐added foods as compared to untaxed sugar‐added foods.

We could not pool any study results or combine intervention groups in a meta‐analysis. On the individual level, the clinical significance of the results is minimal. We are uncertain about the effectiveness of taxing sugar‐added foods, but if there is evidence for small effects in future updates, taxing sugar‐added foods may be meaningful on the population level and important for public health policy actions. Our results are derived from one single study with very low‐certainty evidence and we have to be cautious with the generalisability of the results, as the results apply to the Hungarian setting and it is unclear whether similar and comparable results could be achieved with the same interventions in other European countries or across the globe. For all results, the certainty of evidence is very low, and therefore, we have to be cautious with interpretations as it is not known whether taxing sugar‐added foods is effective to decrease their consumption or improve health outcomes. Our findings demonstrate the necessity of further research to investigate the effectiveness of taxing unprocessed sugar and sugar‐added foods on consumption, expenditure, and health‐related outcomes. As demonstrated in Figure 4, a large part of the assumed pathways remained empty and we were not able to follow one pathway to the end. In summary, there is insufficient evidence to assess whether the taxation of unprocessed sugar and sugar‐added foods other than SSBs is effective to reduce their consumption, demand and expenditure, to improve health outcomes and to cause a dietary shift in terms of substitution and total energy intake.

### Overall completeness and applicability of evidence

In this review, the current body of evidence is insufficient to adequately address the review's objectives. Existing evidence is derived from one study (Biró 2015) and thus, the evidence is limited with respect to comparability (i.e. countries: limited to Hungary) and generalisability of treatment effects. There is a substantial lack of evidence on the effects of taxing unprocessed sugar as we did not identify any study investigating this kind of intervention and its effects. Evidence is completely lacking on the effects of taxing sugar‐added foods on energy intake, total sales of sugar‐added foods, and health‐related outcomes such as overweight and obesity. Evidence on the effects of taxing sugar‐added foods on their consumption and expenditure is addressed by the included study. However, evidence needs to be improved, particularly as the included results might be biased by co‐interventions and misclassification of products into taxed and untaxed food categories. Furthermore, the Hungarian public health product tax did not cover the taxation of all sugar‐added foods, but only selected foods with high sugar content. As a result of the co‐interventions, we have no evidence on the effects of sugar‐added foods implemented as a standalone intervention. For example, the observed effects on the consumption and substitution of and expenditure on sugar‐added foods may result from the interaction of taxation of foods high in salt, sugar and/or caffeine together (for details of the co‐interventions see Table 6), whereas the net effect of the taxation of foods high in sugar alone ‐ on their consumption and expenditure ‐ could be lower and on the substitution effect potentially higher. Households' consumption and expenditure are based on a monthly consumption diary. Accurately measuring consumption and expenditure is challenging. Diary data were found to be substantially biased by measurement error in recall food expenditure (Brzozowski 2017). The study from Schmidt 2014 suggests that individuals report more transactions in the consumption diary within the first days of the diary period as compared to reports in later diary periods. Similarly, the taxed and untaxed products may have been misclassified. These misclassification biases may have affected the results, with underestimation of the effect size likely. For the reasons outlined, evidence from this study may have limited applicability.

### Quality of the evidence

For the taxation of sugar‐added foods, we assessed the certainty of evidence of consumption, substitution and expenditure outcomes as very low. Therefore, the real effect of taxing sugar‐added foods may differ substantially from the estimated effects and thus, our confidence in the effect estimates is very low. Further studies are likely to change the effect estimates of all outcomes included in this review.

We downgraded the maximum of two levels for risk of bias because the study design is an observational study (downgraded once, to 'low', for all outcomes) with a simultaneous intervention of other taxes and likely misclassification of food products as to whether taxed or untaxed (further downgraded to 'very low' for all outcomes). Although it is only possible to downgrade two levels, we could also have downgraded two levels for indirectness because the Hungarian public health product tax is related to specific sugar contents in the particular food categories that were taxed, and the study measured purchased quantities and not consumption; thus, it is not a direct representation of the effect of a complete tax on all sugar‐added foods. The downgrade in this category would apply to all outcomes. We did not downgrade for imprecision. We considered the study to be precise because it is large enough and confidence intervals did not include conflicting values.

### Potential biases in the review process

We rated the risk of potential bias in the review process as low. We have strong confidence that we identified all eligible studies for inclusion in this review. We applied a very broad search strategy for three systematic reviews conducted in tandem, including the taxation of SSBs (Heise 2016), and saturated fat (Lhachimi 2016). The large‐scale search was conducted in 12 electronic databases, six grey literature databases, internet search engines, and key organisational and institutional websites, and was supplemented by handsearching of reference lists to ensure that we identified all potentially relevant records. An Information Specialist (CF), established the search strategy, which a second review author (TLH) partially adapted, and a further Information Specialist reviewed the search strategy on behalf of Cochrane Public Health (Review Milestone 1). Our Information Specialist (CF) or another review author (TLH) conducted all database searches. We also asked our review advisory group members for relevant published and unpublished records. A minimum of two review authors independently screened titles, abstracts and full texts, extracted data and assessed quality (GRADE). We included a broad range of study designs to maximise the completeness of the evidence. In the section Differences between protocol and review, we describe changes between the protocol and review that may have introduced bias. However, in this review, we made no major changes and thus, we prevented reporting bias.

### Agreements and disagreements with other studies or reviews

We are not aware of any previously conducted systematic reviews on the effects of taxing unprocessed sugar or sugar‐added foods on our a priori‐defined primary and secondary outcomes. However, systematic reviews on general food taxes and subsidies suggest a positive effect on consumption, body weight and disease incidence, with greater effects in higher tax rates (e.g. Afshin 2017; Alagiyawanna 2015; Maniadakis 2013; Niebylski 2015; Thow 2011; Thow 2014). Niebylski 2015 suggests a minimum tax of 10% to 15% on different foods and beverages for their effectiveness on consumption and public health. Maniadakis 2013 concludes that effects of food taxes on total caloric intakes might be much smaller. A review of simulation studies suggests that taxes on SSBs and saturated fat and subsidies on healthy foods result in a reduced calorie intake and a decreased consumption of the taxed food (Eyles 2012). However, the evidence base in the mentioned reviews is of low quality with high heterogeneity with respect to tax rates, taxed items, and study designs of included studies. As the interventions in existing reviews and the methodological approaches are completely different to our review, we cannot compare our findings with those of the reviews mentioned above.

## Authors' conclusions

**Implications for practice**

Due to very limited and very low‐certainty evidence, we cannot derive concrete conclusions on the effectiveness of taxing sugar‐added foods for reducing their consumption and preventing obesity or other adverse health outcomes. Despite the reported reduction of the consumption of sugar‐added foods in the primary study, we are uncertain whether taxing sugar‐added foods has an effect on reducing their consumption and preventing obesity or other adverse health outcomes as the evidence is very low certainty. We did not find any studies that looked at the effectiveness of taxing unprocessed sugar for reducing its consumption and preventing obesity or other adverse health outcomes. We did not find any studies that looked at the effects of taxing sugar‐added foods for consumption of unprocessed sugar, energy intake, overweight, and obesity or any health‐related outcomes that would be of great interest to derive implications for practice.

**Implications for research**

Further studies supporting greater certainty of the evidence are required to assess the effectiveness of taxing unprocessed sugar or sugar‐added foods for reducing their consumption and preventing obesity or other adverse health outcomes. To our knowledge, taxes on unprocessed sugar or sugar‐added foods are currently implemented in the following seven countries: Bermuda (first tax implementation on 1 October 2018, revised since 1 April 2019), Dominica (tax implementation on 1 September 2015), Hungary (tax implementation on 1 September 2011), India (tax implementation on 1 July 2017), Norway (tax implementation in 2017, revised since January 2018), St. Vincent and the Grenadines (tax implementation on 1 May 2016), and Navajo Nations, USA (tax implementation on 11 April 2015). Most of these taxes were implemented relatively recently and therefore, there is great potential for further studies on the effects of taxing unprocessed sugar or sugar‐added foods.

Further research is particularly needed in these countries to assess the effects of taxes on unprocessed sugar or sugar‐added foods. Specifically countries that tax unprocessed sugar are of high interest for this review as the taxation of unprocessed sugar affects all other products with sugar as an ingredient in processed foods. Therefore, further studies on the effects of taxing unprocessed sugar, and possibly simultaneously sugar‐added foods, should focus on the taxation effects in Norway and on St. Vincent and the Grenadines. All future studies should also consider health effects as relevant outcome domains.

## Appendices

### Appendix 1. MEDLINE search strategy

27 April 2016: 2884 records

6 December 2016: 336 records

12 January 2018: 374 records

12 September 2019: 641 records

Total: 4235 records

1. exp Taxes/

2. exp Government Programs/ec, lj [Economics, Legislation & Jurisprudence]

3. exp Health Policy/ec, lj [Economics, Legislation & Jurisprudence]

4. exp Food Dispensers, Automatic/ec, lj, sn [Economics, Legislation & Jurisprudence, Statistics & Numerical Data]

5. exp Health Promotion/ec, lj [Economics, Legislation & Jurisprudence]

6. exp Nutrition Policy/ec, lj [Economics, Legislation & Jurisprudence]

7. exp Public Health/ec, lj [Economics, Legislation & Jurisprudence]

8. "demand elasticity".tw.

9. "policy intervention*".tw.

10. "sales tax".tw.

11. "thin subsidies".tw.

12. "vending machine*".tw.

13. budget.tw.

14. excise.tw.

15. fiscal.tw.

16. levied.tw.

17. levy.tw.

18. price.tw.

19. priced.tw.

20. prices.tw.

21. pricing.tw.

22. subsidy.tw.

23. subsidies.tw.

24. tax.tw.

25. taxation.tw.

26. taxed.tw.

27. taxes.tw.

28. taxing.tw.

29. OR/1‐28

30. exp Dietary Carbohydrates/

31. exp Dietary Sucrose/

32. exp High Fructose Corn Syrup/

33. "chewing gum".tw.

34. "dietary sucrose".tw.

35. (("energy dens*" or "highenergy" or "high energy" or "high‐energy" or "low energy" or chips) and (fat* or sugar* or sweet* or food or diet* or nutrition or overweight or drink* or beverage* or protein* or carbohydrate*)).tw.

36. "HED calori*".tw.

37. "HED‐calori*".tw.

38. "highcalori* food*".tw.

39. "high calori* food*".tw.

40. "high‐calori* food*".tw.

41. "lowcalori* food*".tw.

42. "low calori* food*".tw.

43. "low‐calori* food*".tw.

44. "ice cream*".tw.

45. "unhealthy food*".tw.

46. bakery.tw.

47. biscuit*.tw.

48. cacao.tw.

49. cake*.tw.

50. calorie*.tw.

51. candy.tw.

52. candies.tw.

53. bonbon*.tw.

54. chocolate*.tw.

55. confectionar*.tw.

56. cookie*.tw.

57. isoglucose.tw.

58. jam.tw.

59. jelly.tw.

60. jellies.tw.

61. liquorice.tw.

62. macronutrient*.tw.

63. maltose.tw.

64. marmalade.tw.

65. marzipan.tw.

66. pastr*.tw.

67. sucrose.tw.

68. sugar.tw.

69. sugars.tw.

70. sugary.tw.

71. sweet*.tw.

72. exp Butter/

73. exp Dietary Fats/

74. exp Energy Intake/

75. exp Fast Foods/

76. exp Margarine/

77. exp Plant Oils/ec [Economics]

78. "fastfood*".tw.

79. "fast food*".tw.

80. "fast‐food*".tw.

81. "fattening‐food*".tw.

82. "fattening food*".tw.

83. "fried food*".tw.

84. (coconut OR cooking OR palm OR vegetable OR soya OR soybean OR rapeseed OR linseed OR sunflower OR sesame OR peanut OR groundnut OR copra OR babassu OR olive OR thistle ADJ Oil).tw.

85. "salty‐snack*".tw.

86. "salty snack*".tw.

87. "snack food*".tw.

88. "snack‐food*".tw.

89. "takeaway food*".tw.

90. "takeaway‐food*".tw.

91. "take away food*".tw.

92. "take away‐food*".tw.

93. "take‐away food*".tw.

94. "take‐away‐food*".tw.

95. "whole milk".tw.

96. burger*.tw.

97. butter.tw.

98. cheese.tw.

99. cream.tw.

100. crisps.tw.

101. (egg AND (fat* or sugar* or sweet* or food or diet* or nutrition or overweight or drink* or beverage* or protein* or carbohydrate*)).tw.

102. (eggs AND (fat* or sugar* or sweet* or food or diet* or nutrition or overweight or drink* or beverage* or protein* or carbohydrate*)).tw.

103. (fat AND (Food* or diet* or nutrition or nutrient or eat* or meal* or oil* or carbohydrate* or protein* or obesity or obese)).tw.

104. (fatty AND (Food* or diet* or nutrition or nutrient or eat* or meal* or oil* or carbohydrate* or protein* or obesity or obese)).tw.

105. fats.tw.

106. fattening.tw.

107. fries.tw.

108. ghee.tw.

109. lard.tw.

110. margarine.tw.

111. mono‐unsat*.tw.

112. monounsat*.tw.

113. omega3.tw.

114. "omega 3".tw.

115. omega‐3.tw.

116. pizza.tw.

117. polyunsat*.tw.

118. poly‐unsat*.tw.

119. sausage*.tw.

120. suet.tw.

121. exp Carbonated Beverages/

122. exp Food Preferences/

123. exp Food Habits/

124. "caloric‐drink*".tw.

125. "caloric drink*".tw.

126. "carbonated‐beverage*".tw.

127. "carbonated beverage*".tw.

128. "carbonated‐drink*".tw.

129. "carbonated drink*".tw.

130. "energy‐drink*".tw.

131. "energy drink*".tw.

132. "fizzy‐drink*".tw.

133. "fizzy drink*".tw.

134. "high‐calori* drink*".tw.

135. "high calori* drink*".tw.

136. "soda pop".tw.

137. "soft‐drink*".tw.

138. "soft drink*".tw.

139. "sport‐drink*".tw.

140. "sport* drink*".tw.

141. "sport*‐drink*".tw.

142. cola.tw.

143. soda.tw.

144. SSB*.tw.

145. syrup*.tw.

146. OR/30‐145

147. 29 AND 146

148. (animals NOT (humans AND animals)).sh.

149. 147 NOT 148

### Appendix 2. Search strategies for electronic academic databases

#### Cochrane Central Register of Controlled Trials (CENTRAL; 2019, Issue 10) via Wiley (searched 9 October 2019)

19 April 2016: 294 records

7 December 2016: 12 records

19 January 2018: 26 records

9 October 2019: 93

Total: 425 records

#1. MeSH descriptor: [Taxes] explode all trees

#2. MeSH descriptor: [Government Programs] explode all trees and with qualifier(s): [Economics ‐ EC, Legislation & jurisprudence ‐ LJ]

#3. MeSH descriptor: [Health Policy] explode all trees and with qualifier(s): [Economics ‐ EC, Legislation & jurisprudence ‐ LJ]

#4. MeSH descriptor: [Food Dispensers, Automatic] explode all trees

#5. MeSH descriptor: [Health Promotion] explode all trees and with qualifier(s): [Economics ‐ EC, Legislation & jurisprudence ‐ LJ]

#6. MeSH descriptor: [Nutrition Policy] explode all trees and with qualifier(s): [Economics ‐ EC, Legislation & jurisprudence ‐ LJ]

#7. MeSH descriptor: [Public Health] explode all trees and with qualifier(s): [Economics ‐ EC, Legislation & jurisprudence ‐ LJ]

#8. "demand elasticity"

#9. "policy intervention*"

#10. "thin subsidies"

#11. "vending machine*"

#12. budget

#13. excise

#14. fiscal

#15. levied

#16. levy

#17. price

#18. priced

#19. prices

#20. pricing

#21. subsidy

#22 .subsidies

#23 .tax

#24. taxation

#25. taxed

#26. taxes

#27. taxing

#28. #1 or #2 or #3 or #4 or #5 or #6 or #7 or #8 or #9 or #10 or #11 or #12 or #13 or #14 or #15 or #16 or #17 or #18 or #19 or #20 or #21 or #22 or #23 or #24 or #25 or #26 or #27

#29. MeSH descriptor: [Dietary Carbohydrates] explode all trees

#30. MeSH descriptor: [Dietary Sucrose] explode all trees

#31. "chewing gum"

#32. "dietary sucrose"

#33. "energy dens*"

#34. "highenergy"

#35. "high energy"

#36. "high‐energy"

#37. "low energy"

#38. chips

#39. "highcalori* food*"

#40. "high calori* food*"

#41. "high‐calori* food*"

#42. "low‐calori* food*"

#43. "ice cream*"

#44. "unhealthy food*"

#45. bakery

#46. biscuit*

#47. cacao

#48. cake*

#49. calorie*

#50. candy

#51. candies

#52. bonbon*

#53. chocolate*

#54. confectionar*

#55. cookie*

#56. isoglucose

#57. jam

#58. jelly

#59. jellies

#60. liquorice

#61. macronutrient*

#62. maltose

#63. marmalade

#64. marzipan

#65. pastr*

#66. sucrose

#67. sugar

#68. sugars

#69. sugary

#70. sweet*

#71. MeSH descriptor: [Butter] explode all trees

#72. MeSH descriptor: [Dietary Fats] explode all trees

#73. MeSH descriptor: [Energy Intake] explode all trees

#74. MeSH descriptor: [Fast Foods] explode all trees

#75. MeSH descriptor: [Margarine] explode all trees

#76. MeSH descriptor: [Plant Oils] explode all trees

#77. "fastfood*"

#78. "fast food*"

#79. "fast‐food*"

#80. "fattening‐food*"

#81. "fattening food*"

#82. "fried food*"

#83. "coconut oil"

#84. "cooking oil"

#85. "palm oil"

#86. "vegetable oil"

#87. "soya oil"

#88. "soybean oil"

#89. "rapeseed oil"

#90. "linseed oil"

#91. "sunflower oil"

#92. "sesame oil"

#93. "peanut oil"

#94. "groundnut oil"

#95. "copra oil"

#96. "babassu oil"

#97. "olive oil"

#98. "thistle oil"

#99. "salty‐snack*"

#100. "salty snack*"

#101. "snack food*"

#102. "snack‐food*"

#103. "takeaway food*"

#104. "takeaway‐food*"

#105. "take away food*"

#106. "take away‐food*"

#107. "take‐away food*"

#108. "take‐away‐food*"

#109. "whole milk"

#110. burger*

#111. butter

#112. cream

#113. crisps

#114. egg

#115. eggs

#116. fat

#117. fatty

#118. fats

#119. fries

#120. lard

#121. mono‐unsat*

#122. monounsat*

#123. omega3

#124. "omega 3"

#125. omega‐3

#126. polyunsat*

#127. poly‐unsat*

#128. sausage*

#129. suet

#130. MeSH descriptor: [Carbonated Beverages] explode all trees

#131. MeSH descriptor: [Food Preferences] explode all trees

#132. MeSH descriptor: [Food Habits] explode all trees

#133. "caloric‐drink*"

#134. "caloric drink*"

#135. "carbonated‐beverage*"

#136. "carbonated beverage*"

#137. "carbonated‐drink*"

#138. "carbonated drink*"

#139. "energy‐drink*"

#140. "energy drink*"

#141. "fizzy‐drink*"

#142. "fizzy drink*"

#143. "high‐calori* drink*"

#144. "high calori* drink*"

#145. "soda pop"

#146. "soft‐drink*"

#147. "soft drink*"

#148. "sport‐drink*"

#149. "sport* drink*"

#150. "sport*‐drink*"

#151. cola

#152. soda

#153. SSB*

#154. syrup*

#155. #29 or #30 or #31 or #32 or #33 or #34 or #35 or #36 or #37 or #38 or #39 or #40 or #41 or #42 or #43 or #44 or #45 or #46 or #47 or #48 or #49 or #50 or #51 or #52 or #53 or #54 or #55 or #56 or #57 or #58 or #59 or #60 or #61 or #62 or #63 or #64 or #65 or #66 or #67 or #68 or #69 or #70 or #71 or #72 or #73 or #74 or #75 or #76 or #77 or #78 or #79 or #80 or #81 or #82 or #83 or #84 or #85 or #86 or #87 or #88 or #89 or #90 or #91 or #92 or #93 or #94 or #95 or #96 or #97 or #98 or #99 or #100 or #101 or #102 or #103 or #104 or #105 or #106 or #107 or #108 or #109 or #110 or #111 or #112 or #113 or #114 or #115 or #116 or #117 or #118 or #119 or #120 or #121 or #122 or #123 or #124 or #125 or #126 or #127 or #128 or #129 or #130 or #131 or #132 or #133 or #134 or #135 or #136 or #137 or #138 or #139 or #140 or #141 or #142 or #143 or #144 or #145 or #146 or #147 or #148 or #149 or #150 or #151 or #152 or #153 or #154

#156. #155 and #28 in Trials

#### Cochrane Database of Systematic Reviews (CDSR) via Wiley (1995 to present)

19 April 2016: 35 records

7 December 2016: 4 records

19 January 2018: 26 records

9 October 2019: 1 record

Total: 66 records

#1 tax or taxes or taxation

#2 food or sugar or sweet or sweets or sweetened or fast food or snacks or fat or fats or fatty or soft drinks

#3 #1 and #2

#### Excerpta Medica database (Embase) via OvidSP (1947 to present)

12 April 2016: 4633 records

6 December 2016: 469 records

12 January 2018: 672 records

12 September 2019: 1023 records

Total: 6797 records

1. exp Tax/

2. exp government regulation/

3. "demand elasticity".tw.

4. "policy intervention*".tw.

5. "sales tax".tw.

6. "thin subsidies".tw.

7. "vending machine*".tw.

8. budget.tw.

9. excise.tw.

10. fiscal.tw.

11. levied.tw.

12. levy.tw.

13. price.tw.

14. priced.tw.

15. prices.tw.

16. pricing.tw.

17. subsidy.tw.

18. subsidies.tw.

19. tax.tw.

20. taxation.tw.

21. taxed.tw.

22. taxes.tw.

23. taxing.tw.

24. or/1‐23

25. exp carbohydrate intake/

26. exp corn syrup/

27. sugar intake/

28. sweetening agent/

29. "chewing gum".tw.

30. "dietary sucrose".tw.

31. (("energy dens*" or "highenergy" or "high energy" or "high‐energy" or "low energy" or chips) and (fat* or sugar* or sweet* or food or diet* or nutrition or overweight or drink* or beverage* or protein* or carbohydrate*)).tw.

32. "HED calori*".tw.

33. "HED‐calori*".tw.

34. "highcalori* food*".tw.

35. "high calori* food*".tw.

36. "high‐calori* food*".tw.

37. "lowcalori* food*".tw.

38. "low calori* food*".tw.

39. "low‐calori* food*".tw.

40. "ice cream*".tw.

41. "unhealthy food*".tw.

42. bakery.tw.

43. biscuit*.tw.

44. cacao.tw.

45. cake*.tw.

46. calorie*.tw.

47. candy.tw.

48. candies.tw.

49. bonbon*.tw.

50. chocolate*.tw.

51. confectionar*.tw.

52. cookie*.tw.

53. isoglucose.tw.

54. jam.tw.

55. jelly.tw.

56. jellies.tw.

57. liquorice.tw.

58. macronutrient*.tw.

59. maltose.tw.

60. marmalade.tw.

61. marzipan.tw.

62. pastr*.tw.

63. sucrose.tw.

64. sugar.tw.

65. sugars.tw.

66. sugary.tw.

67. sweet*.tw.

68. exp butter/

69. exp fat intake/

70. exp caloric intake/

71. exp fast food/

72. exp margarine/

73. exp food preference/

74. exp milk fat/

75. "fastfood*".tw.

76. "fast food*".tw.

77. "fast‐food*".tw.

78. "fattening‐food*".tw.

79. "fattening food*".tw.

80. "fried food*".tw.

81. ((coconut or cooking or palm or vegetable or soya or soybean or rapeseed or linseed or sunflower or sesame or peanut or groundnut or copra or babassu or olive or thistle) adj Oil).tw.

82. "salty‐snack*".tw.

83. "salty snack*".tw.

84. "snack food*".tw.

85. "snack‐food*".tw.

86. "takeaway food*".tw.

87. "takeaway‐food*".tw.

88. "take away food*".tw.

89. "take away‐food*".tw.

90. "take‐away food*".tw.

91. "take‐away‐food*".tw.

92. "whole milk".tw.

93. burger*.tw.

94. butter.tw.

95. cheese.tw.

96. cream.tw.

97. crisps.tw.

98. (egg and (fat* or sugar* or sweet* or food or diet* or nutrition or overweight or drink* or beverage* or protein* or carbohydrate*)).tw.

99. (eggs and (fat* or sugar* or sweet* or food or diet* or nutrition or overweight or drink* or beverage* or protein* or carbohydrate*)).tw.

100. (fat and (Food* or diet* or nutrition or nutrient or eat* or meal* or oil* or carbohydrate* or protein* or obesity or obese)).tw.

101. (fatty and (Food* or diet* or nutrition or nutrient or eat* or meal* or oil* or carbohydrate* or protein* or obesity or obese)).tw.

102. fats.tw.

103. fattening.tw.

104. fries.tw.

105. ghee.tw.

106. lard.tw.

107. margarine.tw.

108. mono‐unsat*.tw.

109. monounsat*.tw.

110. omega3.tw.

111. "omega 3".tw.

112. omega‐3.tw.

113. pizza.tw.

114. polyunsat*.tw.

115. poly‐unsat*.tw.

116. sausage*.tw.

117. suet.tw.

118. "caloric‐drink*".tw.

119. "caloric drink*".tw.

120. "carbonated‐beverage*".tw.

121. "carbonated beverage*".tw.

122. "carbonated‐drink*".tw.

123. "carbonated drink*".tw.

124. "energy‐drink*".tw.

125. "energy drink*".tw.

126. "fizzy‐drink*".tw.

127. "fizzy drink*".tw.

128. "high‐calori* drink*".tw.

129. "high calori* drink*".tw.

130. "soda pop".tw.

131. "soft‐drink*".tw.

132. "soft drink*".tw.

133. "sport‐drink*".tw.

134. "sport* drink*".tw.

135. "sport*‐drink*".tw.

136. cola.tw.

137. soda.tw.

138. SSB*.tw.

139. syrup*.tw.

140. exp dietary intake/

141. or/25‐140

142. 24 and 141

#### PsycINFO via OvidSP (1887 to present)

13 April 2016: 1336 records

7 December 2016: 308 records

12 January 2018: 145 records

9 October 2019: 189 records

Total: 1978 records

1. exp Taxation/

2. exp Policy Making/

3. exp Government Programs/

4. exp Government Policy Making/

5. "demand elasticity".tw.

6. "policy intervention*".tw.

7. "sales tax".tw.

8. "thin subsidies".tw.

9. "vending machine*".tw.

10. budget.tw.

11. excise.tw.

12. fiscal.tw.

13. levied.tw.

14. levy.tw.

15. price.tw.

16. priced.tw.

17. prices.tw.

18. pricing.tw.

19. subsidy.tw.

20. subsidies.tw.

21. tax.tw.

22. taxation.tw.

23. taxed.tw.

24. taxes.tw.

25. taxing.tw.

26. or/1‐25

27. exp Carbohydrates/

28. exp Food Intake/

29. exp Sugars/

30. "chewing gum".tw.

31. "dietary sucrose".tw.

32. (("energy dens*" or "highenergy" or "high energy" or "high‐energy" or "low energy" or chips) and (fat* or sugar* or sweet* or food or diet* or nutrition or overweight or drink* or beverage* or protein* or carbohydrate*)).tw.

33. "HED calori*".tw.

34. "HED‐calori*".tw.

35. "highcalori* food*".tw.

36. "high calori* food*".tw.

37. "high‐calori* food*".tw.

38. "lowcalori* food*".tw.

39. "low calori* food*".tw.

40. "low‐calori* food*".tw.

41. "ice cream*".tw.

42. "unhealthy food*".tw.

43. bakery.tw.

44. biscuit*.tw.

45. cacao.tw.

46. cake*.tw.

47. calorie*.tw.

48. candy.tw.

49. candies.tw.

50. bonbon*.tw.

51. chocolate*.tw.

52. confectionar*.tw.

53. cookie*.tw.

54. isoglucose.tw.

55. jam.tw.

56. jelly.tw.

57. jellies.tw.

58. liquorice.tw.

59. macronutrient*.tw.

60. maltose.tw.

61. marmalade.tw.

62. marzipan.tw.

63. pastr*.tw.

64. sucrose.tw.

65. sugar.tw.

66. sugars.tw.

67. sugary.tw.

68. sweet*.tw.

69. exp Eating Behavior/

70. exp Fast Food/

71. exp Fatty Acids/

72. "fastfood*".tw.

73. "fast food*".tw.

74. "fast‐food*".tw.

75. "fattening‐food*".tw.

76. "fattening food*".tw.

77. "fried food*".tw.

78. ((coconut or cooking or palm or vegetable or soya or soybean or rapeseed or linseed or sunflower or sesame or peanut or groundnut or copra or babassu or olive or thistle) adj Oil).tw.

79. "salty‐snack*".tw.

80. "salty snack*".tw.

81. "snack food*".tw.

82. "snack‐food*".tw.

83. "takeaway food*".tw.

84. "takeaway‐food*".tw.

85. "take away food*".tw.

86. "take away‐food*".tw.

87. "take‐away food*".tw.

88. "take‐away‐food*".tw.

89. "whole milk".tw.

90. burger*.tw.

91. butter.tw.

92. cheese.tw.

93. cream.tw.

94. crisps.tw.

95. (egg and (fat* or sugar* or sweet* or food or diet* or nutrition or overweight or drink* or beverage* or protein* or carbohydrate*)).tw.

96. (eggs and (fat* or sugar* or sweet* or food or diet* or nutrition or overweight or drink* or beverage* or protein* or carbohydrate*)).tw.

97. (fat and (Food* or diet* or nutrition or nutrient or eat* or meal* or oil* or carbohydrate* or protein* or obesity or obese)).tw.

98. (fatty and (Food* or diet* or nutrition or nutrient or eat* or meal* or oil* or carbohydrate* or protein* or obesity or obese)).tw.

99. fats.tw.

100. fattening.tw.

101. fries.tw.

102. ghee.tw.

103. lard.tw.

104. margarine.tw.

105. mono‐unsat*.tw.

106. monounsat*.tw.

107. omega3.tw.

108. "omega 3".tw.

109. omega‐3.tw.

110. pizza.tw.

111. polyunsat*.tw.

112. poly‐unsat*.tw.

113. sausage*.tw.

114. suet.tw.

115. exp "Beverages (Nonalcoholic)"/

116. exp Food Preferences/

117. "caloric‐drink*".tw.

118. "caloric drink*".tw.

119. "carbonated‐beverage*".tw.

120. "carbonated beverage*".tw.

121. "carbonated‐drink*".tw.

122. "carbonated drink*".tw.

123. "energy‐drink*".tw.

124. "energy drink*".tw.

125. "fizzy‐drink*".tw.

126. "fizzy drink*".tw.

127. "high‐calori* drink*".tw.

128. "high calori* drink*".tw.

129. "soda pop".tw.

130. "soft‐drink*".tw.

131. "soft drink*".tw.

132. "sport‐drink*".tw.

133. "sport* drink*".tw.

134. "sport*‐drink*".tw.

135. cola.tw.

136. soda.tw.

137. SSB*.tw.

138. syrup*.tw.

139. or/27‐138

140. 26 and 139

#### Current Contents Medicine Database of German and German‐Language Journals (CCMed) via LIVIVO (2000 to present)

10 October 2019: 39 records (no previous searches)

Total: 39 records

(((tax OR taxes OR taxation) AND (food OR sugar OR sweet OR sweets OR sweetened OR "fast food" OR snacks OR fat OR fats OR fatty OR "soft drinks" OR "soft drink")) OR ((Steuer OR Steuern OR Besteuerung) AND (Essen OR Lebensmittel OR Zucker OR Süßigkeit OR Süßigkeiten OR gesüßt OR Fastfood OR Snacks OR Fett OR Fette OR fetthaltig OR Süßgetränk OR Süßgetränke OR Softdrink OR Softdrinks))) DB=CCMED

#### Latin American and Caribbean Health Sciences (LILACS) via BIREME/VHL (1982 to present)

19 April 2016: 82 records

6 December 2016: 2 records

12 January 2018: 4 records

12 September 2019: 7 records

Total: 95 records

tax or taxes or taxation or policy making [Words] and food or sugar or sweet or sweets or sweetened or fast food or snacks or fat or fats or fatty or soft drinks [Words]

#### EconLit via EBSCO (1969 to present)

18 April 2016: 82 records

6 December 2016: 108 records

12 January 2018: 425 records

9 October 2019: 267 records

Total: 4142 records

S1. SU Taxes

S2. SU Government Programs

S3. SU Health Policy

S4. SU Health Promotion

S5. SU Nutrition Policy

S6. SU Public Health

S7. TX "demand elasticity"

S8. TX "policy intervention*"

S9. TX "sales tax"

S10. TX "thin subsidies"

S11. TX "vending machine*"

S12. TX budget

S13. TX excise

S14. TX fiscal

S15. TX levied

S16. TX levy

S17. TX price

S18. TX priced

S19. TX prices

S20. TX pricing

S21. TX subsidy

S22. TX subsidies

S23. TX tax

S24. TX taxation

S25. TX taxed

S26. TX taxes

S27. TX taxing

S28. S1 OR S2 OR S3 OR S4 OR S5 OR S6 OR S7 OR S8 OR S9 OR S10 OR S11 OR S12 OR S13 OR S14 OR S15 OR S16 OR S17 OR S18 OR S19 OR S20 OR S21 OR S22 OR S23 OR S24 OR S25 OR S26 OR S27

S29. TX "chewing gum"

S30. TX "dietary sucrose"

S31. TX (("energy dens*" or "highenergy" or "high energy" or "high‐energy" or "low energy" or chips) and (fat* or sugar* or sweet* or food or diet* or nutrition or overweight or drink* or beverage* or protein* or carbohydrate*))

S32. TX "high calori* food*"

S33. TX "high‐calori* food*"

S34. TX "low calori* food*"

S35. TX "low‐calori* food*"

S36. TX "ice cream*"

S37. TX "unhealthy food*"

S38. TX bakery

S39. TX biscuit*

S40. TX cacao

S41. TX cake*

S42. TX calorie*

S43. TX candy

S44. TX candies

S45. TX bonbon*

S46. TX chocolate*

S47. TX confectionar*

S48. TX cookie*

S49. TX isoglucose

S50. TX jam

S51. TX jelly

S52. TX jellies

S53. TX macronutrient*

S54. TX pastr*

S55. TX sucrose

S56. TX sugar

S57. TX sugars

S58. TX sugary

S59. TX sweet*

S60. SU Butter

S61. SU Fast Foods

S62. TX "fastfood*"

S63. TX "fast food*"

S64. TX "fast‐food*"

S65. TX "fattening‐food*"

S66. TX "fattening food*"

S67. TX "fried food*"

S68. TX "coconut oil"

S69. TX "cooking oil"

S70. TX "palm oil"

S71. TX "vegetable oil"

S72. TX "soya oil"

S73. TX "soybean oil"

S74. TX "rapeseed oil"

S75. TX "linseed oil"

S76. TX "sunflower oil"

S77. TX "peanut oil"

S78. TX "groundnut oil"

S79. TX "olive oil"

S80. TX "salty‐snack*"

S81. TX "salty snack*"

S82. TX "snack food*"

S83. TX "snack‐food*"

S84. TX "take away food*"

S85. TX "take away‐food*"

S86. TX "take‐away food*"

S87. TX "take‐away‐food*"

S88. TX "whole milk"

S89. TX burger*

S90. TX butter

S91. TX cheese

S92. TX cream

S93. TX crisps

S94. TX egg

S95. TX eggs

S96. TX fat

S97. TX fatty

S98. TX fats

S99. TX fattening

S100. TX fries

S101. TX ghee

S102. TX lard

S103. TX margarine

S104. TX monounsat*

S105. TX omega3

S106. TX "omega 3"

S107. TX omega‐3

S108. TX pizza

S109. TX polyunsat*

S110. TX sausage*

S111. TX suet

S112. SU Carbonated Beverages

S113. SU Food Preferences

S114. TX Food Habits

S115. TX "carbonated‐beverage

S116. TX "carbonated beverage*"

S117. TX "energy‐drink*"

S118. TX "energy drink*"

S119. TX "high‐calori* drink*"

S120. TX "high calori* drink*"

S121. TX "soda pop"

S122. TX "soft‐drink*"

S123. TX "soft drink*"

S124. TX "sport* drink*"

S125. TX "sport*‐drink*"

S126. TX cola

S127. TX soda

S128. TX SSB*

S129. TX syrup*

S130. S29 OR S30 OR S31 OR S32 OR S33 OR S34 OR S35 OR S36 OR S37 OR S38 OR S39 OR S40 OR S41 OR S42 OR S43 OR S44 OR S45 OR S46 OR S47 OR S48 OR S49 OR S50 OR S51 OR S52 OR S53 OR S54 OR S55 OR S56 OR S57 OR S58 OR S59 OR S60 OR S61 OR S62 OR S63 OR S64 OR S65 OR S66 OR S67 OR S68 OR S69 OR S70 OR S71 OR S72 OR S73 OR S74 OR S75 OR S76 OR S77 OR S78 OR S79 OR S80 OR S81 OR S82 OR S83 OR S84 OR S85 OR S86 OR S87 OR S88 OR S89 OR S90 OR S91 OR S92 OR S93 OR S94 OR S95 OR S96 OR S97 OR S98 OR S99 OR S100 OR S101 OR S102 OR S103 OR S104 OR S105 OR S106 OR S107 OR S108 OR S109 OR S110 OR S111 OR S112 OR S113 OR S114 OR S115 OR S116 OR S117 OR S118 OR S119 OR S120 OR S121 OR S122 OR S123 OR S124 OR S125 OR S126 OR S127 OR S128 OR S129

S131. S28 AND S130

#### Campbell Library via Campbell Collaboration (2004 to present)

26 April 2016: 23 records

7 December 2016: 0 records

19 January 2018: 0 records

9 October 2019: 0 records

23 records

'tax or taxes or taxation' IN All text and 'food* or sugar* or sweet or sweets or sweetened or fast food or snacks or fat or fats or fatty or soft drinks' in All text

#### Food Science and Technology Abstracts (FSTA) via OvidSP (1969 to present)

15 June 2016: 859 records

14 December 2016: 50 records

7 January 2018: 219 records

14 October 2019: 197 records

Total: 1325 records

1 exp pricing/ or taxation/

2 ("tax" or "taxation" or "taxed" or "taxes" or "taxing" or "levy" or "levied").mp.

3 (("price" or "pricing" or "prices") adj2 "change?").mp.

4 (("price" or "pricing" or "prices") adj2 ("intervention?" or "experiment?")).mp.

5 ("sugar?" or "added food?" or "fat?" or "saturated" or "caloric" or "soft drink?" or "SSB?" or "sweetened beverage?" or "soft drink?" or "carbonated drink?").mp.

6 or/1‐4

7 5 and 6

#### Cumulative Index to Nursing and Allied Health Literature (CINAHL) via EBSCO (1937 to present)

13 June 2016: 2250 records

6 December 2016: 46 records

19 January 2018: 55 records

12 September 2019: 441 records

Total: 2792 records

S2 (MH "Government Programs+")

S3 (MH "Health Promotion+")

S4 (MH "Nutrition Policy+")

S5 TX "demand elasticity" OR TX "policy intervention*" OR TX "sales tax" OR TX "thin subsidies" OR TX "vending machine*" OR TX budget OR TX excise OR TX fiscal OR TX levied OR TX levy OR TX price OR TX priced

S6 TX prices OR TX pricing OR TX subsidy OR TX subsidies OR TX tax OR TX taxation OR TX taxed OR TX taxes OR TX taxing

S7 S1 OR S2 OR S3 OR S4 OR S5 OR S6

S8 (MH "Dietary Carbohydrates+")

S9 (MH "Dietary Sucrose")

S10 (MM "High Fructose Corn Syrup")

S11 TX "chewing gum" OR TX ( (("energy dens*" or "highenergy" or "high energy" or "high‐energy" or "low energy" or chips) and (fat* or sugar* or sweet* or food or diet* or nutrition or overweight or drink* or beverage* or protein* or carbohydrate*)) ) OR TX "high calori* food*" OR TX "high‐calori* food*" OR TX "low calori* food*" OR TX "low‐calori* food*" OR TX "ice cream*" OR TX "unhealthy food*" OR TX bakery OR TX biscuit* OR TX cacao OR TX cake*

S12 TX calorie* OR TX candy OR TX bonbon* OR TX chocolate* OR TX confectionar* OR TX cookie* OR TX isoglucose OR TX jam OR TX jelly OR TX jellies OR TX macronutrient* OR TX pastr*

S13 TX sucrose OR TX sugar OR TX sugars OR TX sugary OR TX sweet* OR TX Butter OR TX "fastfood*" OR TX "fast food*" OR TX "fast‐food*" OR TX "fattening‐food*" OR TX "fattening food*" OR TX "fried food*"

S14 TX "coconut oil" OR TX "cooking oil" OR TX "palm oil" OR TX "vegetable oil" OR TX "soya oil" OR TX "soybean oil" OR TX "rapeseed oil" OR TX "linseed oil" OR TX "sunflower oil" OR TX "peanut oil" OR TX "groundnut oil" OR TX "olive oil"

S15 TX "salty‐snack*" OR TX "salty snack*" OR TX "snack food*" OR TX "snack‐food*" OR TX "take away food" OR TX "take away‐food*" OR TX "take‐away food" OR TX "take‐away‐food*" OR TX "whole milk" OR TX burger* OR TX cheese OR TX cream

S16 TX crisps OR TX ( (egg and (fat* or sugar* or sweet* or food or diet* or nutrition or overweight or drink* or beverage* or protein* or carbohydrate*)) ) OR TX ( (eggs and (fat* or sugar* or sweet* or food or diet* or nutrition or overweight or drink* or beverage* or protein* or carbohydrate*)) ) OR TX ( (fat and (Food* or diet* or nutrition or nutrient or eat* or meal* or oil* or carbohydrate* or protein* or obesity or obese)) ) OR TX ( (fatty and (Food* or diet* or nutrition or nutrient or eat* or meal* or oil* or carbohydrate* or protein* or obesity or obese)) ) OR TX fats OR TX fattening OR TX fries OR TX ghee OR TX lard OR TX margarine OR TX mono‐unsat*

S17 TX monounsat* OR TX omega3 OR TX "omega 3" OR TX omega‐3 OR TX pizza OR TX polyunsat* OR TX sausage* OR TX suet

S18 (MM "Carbonated Beverages")

S19 (MM "Food Preferences")

S20 (MM "Food Habits")

S21 TX "caloric‐drink*" OR TX "caloric drink*" OR TX "carbonated‐beverage*" OR TX carbonated beverages OR TX "carbonated‐drink*".

S22 TX "carbonated drink*" OR TX "energy‐drink*" OR TX "energy drink*" OR TX "fizzy‐drink*" OR TX "fizzy drink*" OR TX "high‐calori* drink*" OR TX "high calori* drink*" OR TX "soda pop" OR TX "soft‐drink*" OR TX "soft drink*" OR TX "sport‐drink*" OR TX "sport* drink*"

S23 TX "sport*‐drink*" OR TX cola OR TX soda OR TX SSB* OR TX syrup*

S24 S8 OR S9 OR S10 OR S11 OR S12 OR S13 OR S14 OR S15 OR S16 OR S17 OR S18 OR S19 OR S20 OR S21 OR S22 OR S23

S25 S7 AND S24

S26 Restrict S25 to Academic Journals and Dissertations

#### Web of Science (SCI‐EXPANDED, SSCI, A&HCI, CPCI‐S, CPCI‐SSH, ESCI, CCR‐EXPANDED, IC) via Clarivate Analytics (1900 to present)

21 June 2016: 748 records

6 December 2016: 107 records

26 January 2018: 166 records

12 September 2019: 343 records

Total: 1364 records

TOPIC: (((("TAX" OR "TAXATION" OR "TAXED" OR "TAXES" OR "TAXING" OR "LEVY" OR "LEVIED") OR (("PRICE" OR "PRICING" OR "PRICES") NEAR "CHANGE$") OR (("PRICE" OR "PRICING" OR "PRICES") NEAR ("INTERVENTION$" OR "EXPERIMENT$"))))) AND TOPIC: ((("SUGAR$" OR "ADDED FOOD$" OR "FAT$" OR "SATURATED" OR "CALORIC" OR "SOFT DRINK$" OR "SSB$" OR "SWEETENED BEVERAGE$" OR "SOFT DRINK$" OR "CARBONATED DRINK$")))

Timespan: All years. Indexes: SCI‐EXPANDED, SSCI, A&HCI, CPCI‐S, CPCI‐SSH, BKCI‐S, BKCI‐SSH, ESCI, CCR‐EXPANDED, IC

### Appendix 3. Search strategies for grey literature databases

#### ProQuest Dissertations & Theses Database (PQDT): UK and Ireland via ProQuest

16 May 2016: 68 records

7 December 2016: 0 records

19 January 2018: 4 records

9 September 2019: 33 records

Total: 105 records

(ab(tax) OR ti(tax) OR ab(taxes) OR ti(taxes) OR ab(taxation) OR ti(taxation) ab(budget*) OR ti(budget*) OR ab(excise) or ti(excise)) AND (ab(sugar*) OR ti(sugar*) OR ab(sweet*) OR ti(sweet*) OR ab("fast food*") OR ti("fast food*") OR ab(snack*) OR ti(snack*) OR ab(fat) OR ti(fat) OR ab(fatty) OR ti(fatty) OR ab(fats) OR ti(fats) OR ab("soft drink*") OR ti("soft drink*") OR ab(beverage*) OR ti(beverage*) OR ab(food*) OR ti(food*))

#### System for Information on Grey Literature in Europe – OpenGrey via OpenGrey

16 May 2016: 33 records

7 December 2016: 0 records

19 January 2018: 0 records

9 September 2019: 0 records

Total: 33 records

((tax OR taxes OR taxation) AND (food OR sugar OR sweet OR sweets OR sweetened OR fast food OR snacks OR fat OR fats OR fatty OR "soft drinks" OR "soft drink"))

#### The Directory of Open Access Repositories – OpenDOAR via OpenDOAR

18 May 2016: 50

12 December 2016: 21 records

Database not accessible in subsequent searches

Total: 71 records

(tax OR taxes OR taxation) AND (food OR sugar OR sweet OR sweets OR sweetened OR fast food OR snacks OR fat OR fats OR fatty OR "soft drinks" OR "soft drink")

#### EconPapers via REPEC

13 June 2016: 50 records

14 December 2016: 6 records

26 January 2018: 11 records

14 October 2019: 23 records

Total: 90 records

(tax OR taxes OR taxation) AND (food OR sugar OR sweet OR sweets OR sweetened OR fast food OR snacks OR fat OR fats OR fatty OR "soft drinks" OR "soft drink")

#### Social Science Research Network – SSRN eLibrary via SSRN

13 June 2016: 80 records

12 December 2016: 7 records

26 January 2018: 37 records

14 October 2019: 44 records

Total: 168 records

"sugar tax" OR sweetened OR "Nutrient‐Specific Taxes" OR "soda taxes" OR "food tax" OR "fat tax"

#### National Bureau of Economic Research (NBER) via NBER

13 June 2016: 50 records

7 December 2016: 16 records

26 January 2018: 89 records

13 October 2019: 180 records

Total: 335 records

(tax OR taxes OR taxation) AND (food OR sugar OR sweet OR sweets OR sweetened OR fast food OR snacks OR fat OR fats OR fatty OR "soft drinks" OR "soft drink")

#### WHO International Clinical Trials Registry Platform (WHO ICTRP) (includes references of the ClinicalTrials.gov database)

11 August 2016: 94 records

14 October 2019: 70 records

Total: 164 records

(TITLE: tax or taxation or taxed or taxes or taxing or levy or levied or price or pricing or prices) OR (INTERVENTION: tax or taxation or taxed or taxes or taxing or levy or levied or price or pricing or prices)

#### Trials Register of Promoting Health Interventions (TRoPHI) via EPPI‐Centre

11 August 2016: 41 records

Free text search not accessible in subsequent searches

Total: 41 records

Freetext (All but Authors): "tax" or "taxation" or "taxed" or "taxes" or "taxing" or "levy" or "levied" or "price" or "pricing" or "prices"

### Appendix 4. Search strategies for internet search engines

#### Google Scholar via Google

11 August 2016: 30 records

14 October 2019: 30 records

Total: 60 records

(tax OR taxes OR taxation) AND (food OR sugar OR sweet OR sweets OR sweetened OR fast food OR snacks OR fat OR fats OR fatty OR "soft drinks" OR "soft drink")

## Characteristics of studies

### Characteristics of included studies [ordered by study ID]

**Table:** Biró 2015

Methods	**Study design:** interrupted time series (ITS) **Study location/setting:** Hungary **Timing:** retrospective **Allocation to group:** study without control group **Number of individuals:** annual sample of around 26,000 individuals **Number of clusters or sites:** annual sample of around 10,000 households, overall 44,608 household‐level observations; each household remains in the sample for four years; each month, 1/12 of the households run a diary of the expenditure throughout the whole month **Database:** Hungarian Household Budget and Living Survey, administered by the Hungarian Central Statistical Office **Year of study:** 2015 **Duration of the study:** January 2008‐December 2012 (5 survey waves) **Pre‐intervention:** January 2008‐August 2011 (44 months) **Intervention:** 1 September 2011, revision on 1 January 2012 **Post‐intervention:** September 2011‐December 2012 (follow‐up: 16 months) **Analysis:** fixed‐effect models of the standardised measures of taxed and untaxed sugar‐added food consumption	
Participants	**Country:** Hungary **Language:** Hungarian **Age:** all ages **Sex:** both sexes **Socioeconomic characteristics:** different socioeconomic groups **Eligibility criteria:** N/A **Inclusion criteria:** the study is based on data from the Hungarian Household Budget and Living Survey, administered by the Hungarian Central Statistical Office. The study's primary report does not offer information on inclusion criteria. **Recruitment:** the study's primary report does not offer information on participants' recruitment. **Equity considerations:** quote: "The estimated effects were driven by households belonging to the lowest income quartile, who are more responsive to increases in price."	
Interventions	**Intervention:** the Hungarian public health product tax was implemented on the national level in Hungary on 1 September 2011 and revised on 1 January 2012. The intervention includes taxes on sugar‐added foods: pre‐packaged products with added sugar, containing sugar contents > 25 g per 100 g; taxation level on 1 September 2011: 100 HUF/kg (around USD 0.34); taxation level on 1 January 2012: 130 HUF/kg (around USD 0.44) chocolates, containing sugar contents > 40 g per 100 g and cocoa content < 40 g per 100 g; taxation level on 1 September 2011: 100 HUF/kg (around USD 0.34); taxation level on 1 January 2012: 130 HUF/kg (around USD 0.44) sugar‐sweetened cocoa powder, containing sugar contents > 40 g per 100 g and cocoa content < 40 g per 100 g; taxation level on 1 September 2011: taxation level on 1 January 2012: 70 HUF/kg (around USD 0.24) jam, containing sugar contents > 35 g per 100 g; taxation level on 1 September 2011: taxation level on 1 January 2012: 500 HUF/kg (around USD 1.68) **Co‐interventions:** the Hungarian public health product tax also includes SSBs, products high in caffeine, and products high in salt: SSBs, containing sugar contents > 8 g sugar/100 mL; taxation level on 1 September 2011: 5 HUF/L (around USD 0.02); taxation level on 1 January 2012: 7 HUF/L (around USD 0.02) SSB concentrates and syrups, containing sugar contents >8 g sugar/100 mL and fruit < 25%; taxation level on 1 September 2011: taxation level on 1 January 2012: 200 HUF/L (around USD 0.67) flavoured beer or alcoholic drink, containing > 5 g sugar/100 mL; taxation level on 1 September 2011: taxation level on 1 January 2012: 20 HUF/L (around USD 0.07) energy drinks, containing > 8 g sugar/100 mL or caffeine > 10 mg/100 mL; taxation level on 1 September 2011: 250 HUF/L (around USD 0.84); taxation level on 1 January 2012 energy drinks, containing > 1 mg methylxanthines/100 mL or > 100 mg taurine/100 mL; taxation level on 1 September 2011: taxation level on 1 January 2012: 250 HUF/L (around USD 0.84) salted snacks, containing > 1 g salt/100 g; taxation level on 1 September 2011: 200 HUF/kg (around USD 0.67); taxation level on 1 January 2012: 250 HUF/kg (around USD 0.84) condiments (some exemptions for mustards, ketchups), containing > 5 g salt/100 g; taxation level on 1 September 2011: 200 HUF/kg (around USD 0.67); taxation level on 1 January 2012: 250 HUF/kg (around USD 0.84)	
Outcomes	**Primary outcomes:** consumption of unprocessed sugar or sugar‐added foods: mean consumption of taxed sugar‐added foods (measured in units of kg), mean consumption of untaxed sugar‐added foods (measured in units of kg), SMD in the consumption of taxed sugar‐added foods vs untaxed sugar‐added foods (measured in units of kg), assessed continuously throughout the 5 study waves with routinely collected data from consumption diaries energy intake: not measured overweight and obesity: not measured **Secondary outcomes:** expenditure: mean expenditure on taxed sugar‐added foods (measured in units of HUF, mean expenditure on untaxed sugar‐added foods (measured in units of HUF), and SMD in the expenditure on taxed sugar‐added foods vs untaxed sugar‐added foods, assessed continuously throughout the 5 study waves with routinely collected data from consumption diaries substitution and diet: not measured demand: not measured other health outcomes: not measured	
Notes	**Sources of funding:** the study was funded by the Scottish Institute for Research in Economics (SIRE) Early Career Engagement Grant	
***Risk of bias***		
**Bias**	**Authors' judgement**	**Support for judgement**
Blinding of participants and personnel (performance bias) All outcomes	Low risk	Comment: although participants and personnel were not blinded and all participants received the intervention, participants and personnel of the Hungarian Household Budget and Living Conditions Survey did not know that data were collected to measure the effect of the Hungarian public health product tax.
Blinding of outcome assessment (detection bias) All outcomes	Low risk	Comment: outcome assessment is based on objective self‐reports
Incomplete outcome data (attrition bias) All outcomes	Unclear risk	Comment: information on how and whether incomplete outcome data were addressed is not reported.
Selective reporting (reporting bias)	Low risk	Comment: supplement gives an overview of the complete data.
Other bias	High risk	Comment: the categorisation of foods into taxed and untaxed food categories may be wrong in parts as relevant information for building correct categories was missing.
Intervention independent	High risk	Comment: the intervention on the taxation of sugar‐added foods was accompanied by the taxation of SSBs and foods high in salt and caffeine. Outcomes might be affected by the other interventions.
Shape of effect pre‐specified	Low risk	Quote from report: "The variable time captures a linear trend measured in months. Vector includes a set of household characteristics. The time‐specific indicator of taxation is Tt=I(T >=September 2011)." (p. 111)
Intervention had no effect on data collection	Low risk	Quote from report: "I use data from the Hungarian Household Budget and Living Conditions Survey. The representative survey is administered by the Hungarian Central Statistical Office, it has been running in its current form since 2009, the data I use correspond to years 2008–2012." (p. 110)

**HUF:** Hungarian Forint; **SMD:** standardised mean difference; **SSB:** sugar‐sweetened beverage

### Characteristics of excluded studies [ordered by study ID]

**Table:**

Study	Reason for exclusion
Appelhans 2018	Intervention not eligible: no intervention on unprocessed sugar or sugar‐added foods
Batis 2016	Intervention not eligible: no intervention on unprocessed sugar or sugar‐added foods, intervention addresses foods with energy density ≥ 250 kcal/100 g
Bridgman 2007	Study design not eligible: simulation study. Intervention not eligible: no intervention on unprocessed sugar or sugar‐added foods. Outcome not eligible: no eligible outcome reported
Elbel 2013	Intervention not eligible: no baseline directly before implementation of the tax intervention conditions 3‐5. The definition of 'healthier' and 'less‐healthier' remains unclear as, "researchers defined items that met at least two of the three standards in their entirety as healthier, and other items as less healthy" (p. 50). Outcomes on sugar contain both foods and beverages together.
Hanks 2013	Intervention not eligible: less healthy foods were taxed, healthier foods were subsidised. Taxation without information on sugar levels. Healthy foods contain sugar‐added foods.
Hanks 2014	Intervention not eligible: less healthy foods were taxed, healthier foods were subsidised. Taxation without information on sugar levels. Healthy foods contain sugar‐added foods.
Martos 2016	Study design not eligible: cross‐sectional study without pre‐intervention baseline. Intervention not eligible: two independent sections after implementation of tax.
Mauricio 2019	Intervention not eligible: no intervention on unprocessed sugar or sugar‐added foods, intervention addresses foods with energy density ≥ 250 kcal/100 g
Taillie 2017	Intervention not eligible: untaxed food contains salty snacks, cereals and sugar‐added foods. Intervention is applied to high caloric foods.
Unar‐Munguia 2019	Intervention not eligible: NAFTA as intervention. No continuity of taxation: simultaneous and overlapping price increases and decreases.

**NAFTA:** North American Free Trade Agreement

## Differences between protocol and review

### Background

We updated the background to outline the current state of research.

### Methods

#### Types of secondary outcomes

We further specified and included the following secondary outcomes.

- Substitution:
  - consumption of untaxed sugar‐added foods;
  - difference in mean consumption of taxed sugar‐added foods compared with untaxed sugar‐added foods.
- Expenditure:
  - expenditure on untaxed sugar‐added foods;
  - difference in mean consumption of taxed sugar‐added foods compared with untaxed sugar‐added foods.

#### Types of interventions

We further specified our eligibility criteria on types of interventions. We excluded virtual and hypothetical interventions imitating a taxation on unprocessed sugar or sugar‐added foods if participants' purchase decisions were not binding so that they did not all result in a real purchase or if the money was virtual or not belonging to the study participant.

#### Searches

We did not search the website from The Obesity Society (www.obesity.org), as it was not possible to enter the search terms. The database from the website The Directory of Open Access Repositories – OpenDOAR via OpenDOAR was not accessible after the search on 12 December 2016. The free text search on the website Trials Register of Promoting Health Interventions (TRoPHI) via EPPI‐Centre was not accessible after the search on 11 August 2016.

#### Data synthesis

We did not undertake harvest plots for narrative synthesis.
